# SK channel-mediated metabolic escape to glycolysis inhibits ferroptosis and supports stress resistance in *C. elegans*

**DOI:** 10.1038/s41419-020-2458-4

**Published:** 2020-04-23

**Authors:** Inge E. Krabbendam, Birgit Honrath, Benjamin Dilberger, Eligio F. Iannetti, Robyn S. Branicky, Tammo Meyer, Bernard Evers, Frank J. Dekker, Werner J. H. Koopman, Julien Beyrath, Daniele Bano, Martina Schmidt, Barbara M. Bakker, Siegfried Hekimi, Carsten Culmsee, Gunter P. Eckert, Amalia M. Dolga

**Affiliations:** 1 Faculty of Science and Engineering, Department of Molecular Pharmacology, Groningen Research Institute of Pharmacy (GRIP), University of Groningen, 9713 AV Groningen, The Netherlands; 2 German Center for Neurodegenerative Diseases (DZNE) e.V., Sigmund-Freud-Straße 27, 53127 Bonn, Germany; 3 Institut für Pharmakologie und Klinische Pharmazie, Biochemisch-Pharmakologisches Centrum Marburg, Philipps-Universität Marburg, Karl-von-Frisch-Straße 2, Marburg, 35032 Germany; 4 Faculty of Agricultural Sciences, Nutritional Sciences, and Environmental Management, Institute of Nutritional Sciences, Justus-Liebig-University of Giessen, 35392 Giessen, Germany; 5 Khondrion, Philips van Leydenlaan 15, 6525EX Nijmegen, The Netherlands; 6 Department of Biology, McGill University, 1205 Ave Docteur Penfield, Montreal, QC H3A 1B1 Canada; 7 Department of Pediatrics, Section Systems Medicine of Metabolism and Signalling, University of Groningen, University Medical Center Groningen, A. Deusinglaan 1, 9713 AV Groningen, The Netherlands; 8 Systems Biology Centre for Energy Metabolism and Ageing, University of Groningen, University Medical Center Groningen, A. Deusinglaan 1, 9713 AV Groningen, The Netherlands; 9 Department of Chemical and Pharmaceutical Biology, Groningen Research Institute of Pharmacy (GRIP), University of Groningen, Groningen, The Netherlands; 10 Radboud University Medical Center, Department of Biochemistry (286), Nijmegen, The Netherlands; 11 Center for Mind Brain and Behavior—CMBB, University of Marburg, Hans-Meerwein-Straße 6, 35032 Marburg, Germany

**Keywords:** Stress signalling, Mechanisms of disease

## Abstract

Metabolic flexibility is an essential characteristic of eukaryotic cells in order to adapt to physiological and environmental changes. Especially in mammalian cells, the metabolic switch from mitochondrial respiration to aerobic glycolysis provides flexibility to sustain cellular energy in pathophysiological conditions. For example, attenuation of mitochondrial respiration and/or metabolic shifts to glycolysis result in a metabolic rewiring that provide beneficial effects in neurodegenerative processes. Ferroptosis, a non-apoptotic form of cell death triggered by an impaired redox balance is gaining attention in the field of neurodegeneration. We showed recently that activation of small-conductance calcium-activated K^+^ (SK) channels modulated mitochondrial respiration and protected neuronal cells from oxidative death. Here, we investigated whether SK channel activation with CyPPA induces a glycolytic shift thereby increasing resilience of neuronal cells against ferroptosis, induced by erastin in vitro and in the nematode *C. elegans* exposed to mitochondrial poisons in vivo. High-resolution respirometry and extracellular flux analysis revealed that CyPPA, a positive modulator of SK channels, slightly reduced mitochondrial complex I activity, while increasing glycolysis and lactate production. Concomitantly, CyPPA rescued the neuronal cells from ferroptosis, while scavenging mitochondrial ROS and inhibiting glycolysis reduced its protection. Furthermore, SK channel activation increased survival of *C. elegans* challenged with mitochondrial toxins. Our findings shed light on metabolic mechanisms promoted through SK channel activation through mitohormesis, which enhances neuronal resilience against ferroptosis in vitro and promotes longevity in vivo.

## Introduction

Metabolic reprogramming towards glycolysis allows for better cellular fitness to sustain cellular energy in situations of scarce nutrient availability, hypoxia, or increased energy demands. Since defects in cellular energy metabolism are established characteristics of neurodegenerative diseases^1–3^, many studies probed for agents with the ability to shift cellular energy metabolism^4,5^. For example, a small molecule screening platform identified the commonly used anti-emetic drug meclizine mediating such metabolic reprogramming and protecting against cell death in models of stroke and Huntington’s disease. Recent findings on cell death mechanisms of neurodegeneration identified ferroptosis, a non-apoptotic form of cell death, as a trigger for the formation of lipid peroxides and also of mitochondrial dysfunction^6–10^. Extensive evidence links ferroptosis to the field of neuroscience with increasing indication for its contribution to brain degeneration, as detected in Alzheimer’s disease (AD)^8,10^. Importantly, inhibitors of ferroptosis were protective in models of neurodegeneration^6,11,12^. Ferroptotic cell death can be initiated by compounds inhibiting the cysteine/glutamate antiporter, such as erastin or glutamate, that mediate depletion of glutathione (GSH), inhibition of glutathione peroxidase 4 (Gpx4)^6,13–15^, and lipid peroxide formation^15,16^. This results in a boost of mitochondrial reactive oxygen species (ROS) to such a level that cellular ROS production exceeds detoxification, causing increased steady-state ROS levels, and culminating in cell death.

Activation of mitochondrial calcium-activated potassium (mitoK_Ca_) channels mediated neuroprotection in different models of oxidative toxicity^17,18^. In particular, activation of small-conductance calcium-activated potassium (SK/K_Ca_) channels preserved cell survival in conditions of oxidative stress and mitochondrial dysfunction^17,19^. In our previous studies, we observed that SK channel activation alone induced a mild increase in mitochondrial ROS levels^19^, leading to the hypothesis that SK channels might render neuroprotective effects via mitochondrial preconditioning or mitohormesis^20–24^. During mitohormesis, cells and tissues undergo adaptation to oxidative stress where mild stress increases the resistance of both mitochondria and the whole cell to subsequent insults^20,25^. This response has been shown to protect against an elevated redox balance in many model organisms. In this regard, in vivo studies in mice and in the nematode *Caenorhabditis elegans* (*C. elegans*) have revealed that non-toxic mitochondrial ROS levels or partially inhibited mitochondrial respiration stimulates pro-longevity programs^26,27^. For example, *C. elegans* mutants with higher mitochondrial ROS levels exhibited an adaptive response by inducing ROS defense enzymes. Elevation of this particular ROS signal was necessary and sufficient to increase lifespan, whereas its inhibition by antioxidants reduced lifespan^28–30^.

To date, potential effects of SK channels on mitohormesis and associated protective effects have not been studied in association with metabolic reprogramming, nor the subsequent effects on survival in whole organisms exposed to conditions of oxidative stress. Therefore, the current study aimed to understand the mechanism by which SK channels confer neuroprotection during ferroptosis and whether metabolic reprogramming contributes to its protective effects.

## Results

### Activation of SK channels preserves cell survival against erastin-induced ferroptosis

Here, we investigated whether the observed neuroprotection was mediated by changes in metabolic programs of cells. To this end, cells were grown in either glucose- or galactose-based medium. Compared to glucose, galactose is used by cells for glycolysis at a slower rate, due to its slow and energy-demanding conversion, thereby increasing the cell’s reliance on OXPHOS for ATP production^31–33^.

**Fig. 1:**
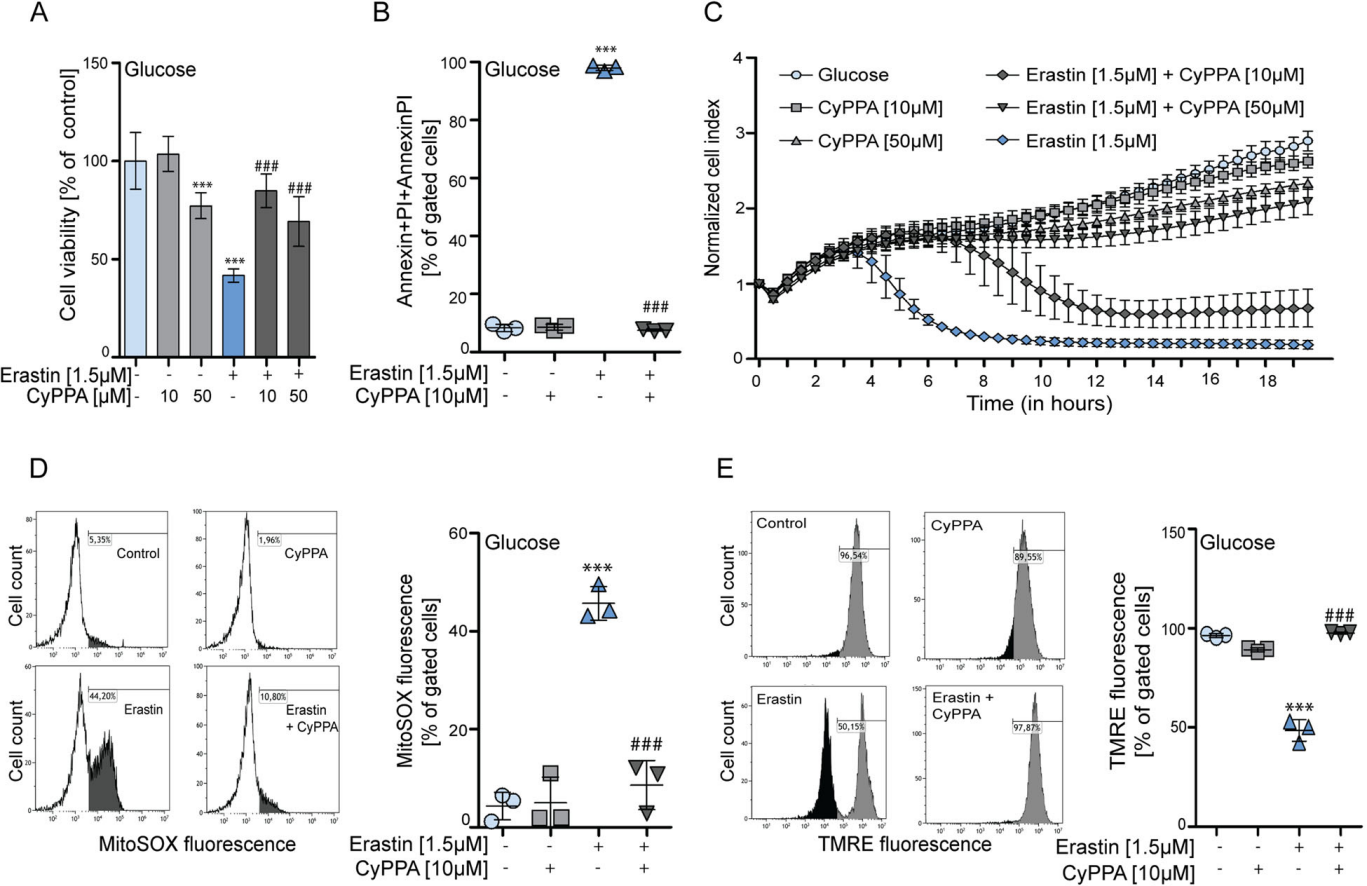
Activation of SK channels preserves cell survival in conditions of erastin-induced ferroptosis. HT22 cells treated with erastin (1.5 µM, 16 h) in the presence or absence of CyPPA (10 µM or 50 µM) in glucose medium. Cell viability was measured by an **a** MTT assay and **b** Annexin V/PI fluorescence by FACS and **c** xCELLigence system. CyPPA treatment is represented as grey curves. Mitochondrial function was assessed by FACS analysis of HT22 cells challenged with erastin (1.5 µM, 16 h) in the presence or absence of CyPPA (10 µM) in glucose. **d** Mitochondrial superoxide levels using MitoSOX and **e** MMP loss using TMRE were determined by FACS analysis. Data are presented as mean ± SD, *n* = 3–6, *p < 0.05, **p < 0.01, ***p < 0.001, ^#^*p* < 0.05, ^##^*p* < 0.01, ^###^*p* < 0.001, *compared to control ^#^compared to erastin alone. All experiments were independently repeated at least three times.

**Fig. 2:**
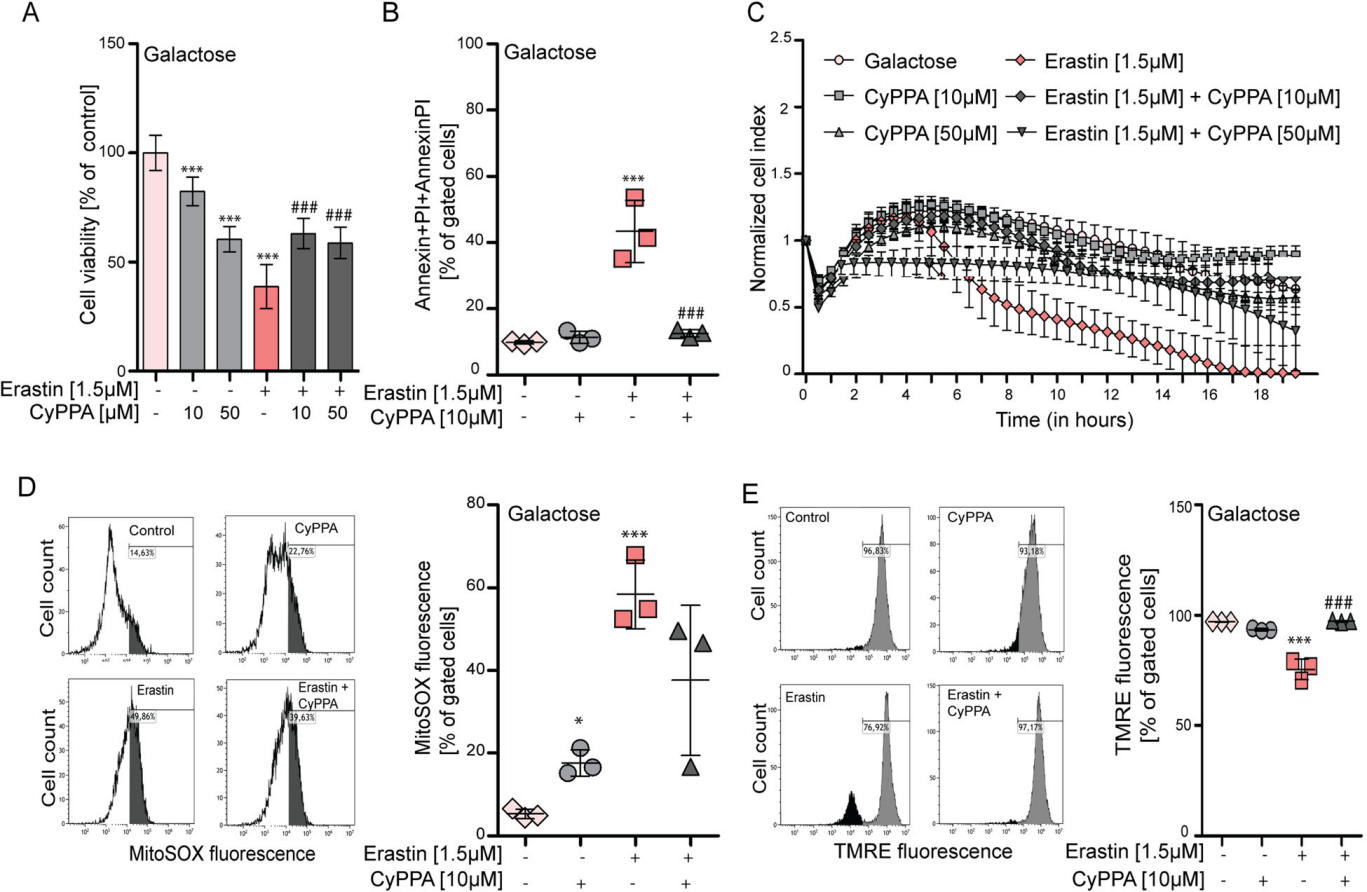
Ferroptotic cell death is prevented by SK channel activation in galactose medium. HT22 cells treated with erastin (1.5 µM, 16 h) in the presence or absence of CyPPA (10 µM or 50 µM) in galactose medium. Cell survival is shown by **a** MTT assay, **b** FACS analysis of Annexin V/PI, and **c** xCELLigence system. Mitochondrial function was assessed by FACS analysis of HT22 cells challenged with erastin (1.5 µM, 16 h) in the presence or absence of CyPPA (10 µM) in glucose. **d** Mitochondrial superoxide levels using MitoSOX and **e** MMP loss using TMRE were determined by FACS analysis. Data are presented as mean ± SD, *n* = 3–6, *p < 0.05, **p < 0.01, ***p < 0.001, ^#^*p* < 0.05, ^##^*p* < 0.01, ^###^*p* < 0.001, *compared to control ^#^compared to erastin alone. All experiments were independently repeated at least three times.

Neuronal HT22 cells represent an established model to study cellular mechanisms related to erastin-induced ferroptotic cell death^34^. Upon application of erastin, cell viability in both glucose- and galactose-based media was reduced (Fig. S1). However, in galactose medium, the effect of erastin on cell viability was less pronounced compared to cells grown in glucose, indicating that ferroptosis induction is accelerated by glucose as carbon substrate for energy generation. Next, we assessed the effects of the SK channel opener CyPPA on erastin-induced toxicity and found that CyPPA prevented cell death, as detected by the MTT metabolic assay (Figs. 1A, 2A, S1), Annexin/PI (Figs. 1B, 2B) and xCELLigence real-time impedance measurements (Figs. 1C, 2C, S1A,C). The MTT assay revealed a reduction of absorbance levels following CyPPA treatment, indicating an effect of SK channel activation on the cellular metabolism. However, analysis of Annexin/PI showed that CyPPA completely prevented ferroptosis-induced cell death. In addition, CyPPA reduced both mitochondrial ROS levels and prevented mitochondrial membrane potential (MMP) loss in erastin-challenged cells (Figs. 1D,E, 2D,E). Notably, erastin-induced toxicity was more severe in glucose as indicated by a stronger loss of the MMP and a stronger increase in mitochondrial ROS levels compared to galactose conditions. The effect of CyPPA on erastin-induced mitochondrial superoxide levels was considerably smaller in galactose medium compared to glucose medium. GSH depletion by BSO had similar effects as erastin on HT22 cells, i.e., cell viability was reduced, as measured by MTT assay, Annexin/PI analysis and real-time impedance measurements (Fig. S2A–C). CyPPA was able to protect against BSO-induced toxicity. Furthermore, mitochondrial ROS levels were strongly increased by BSO challenge and co-treatment with CyPPA was able to prevent the mitochondrial ROS levels (Fig. S2D).

### SK channel activation exerts differential effects in glucose and galactose-based media

**Fig. 3:**
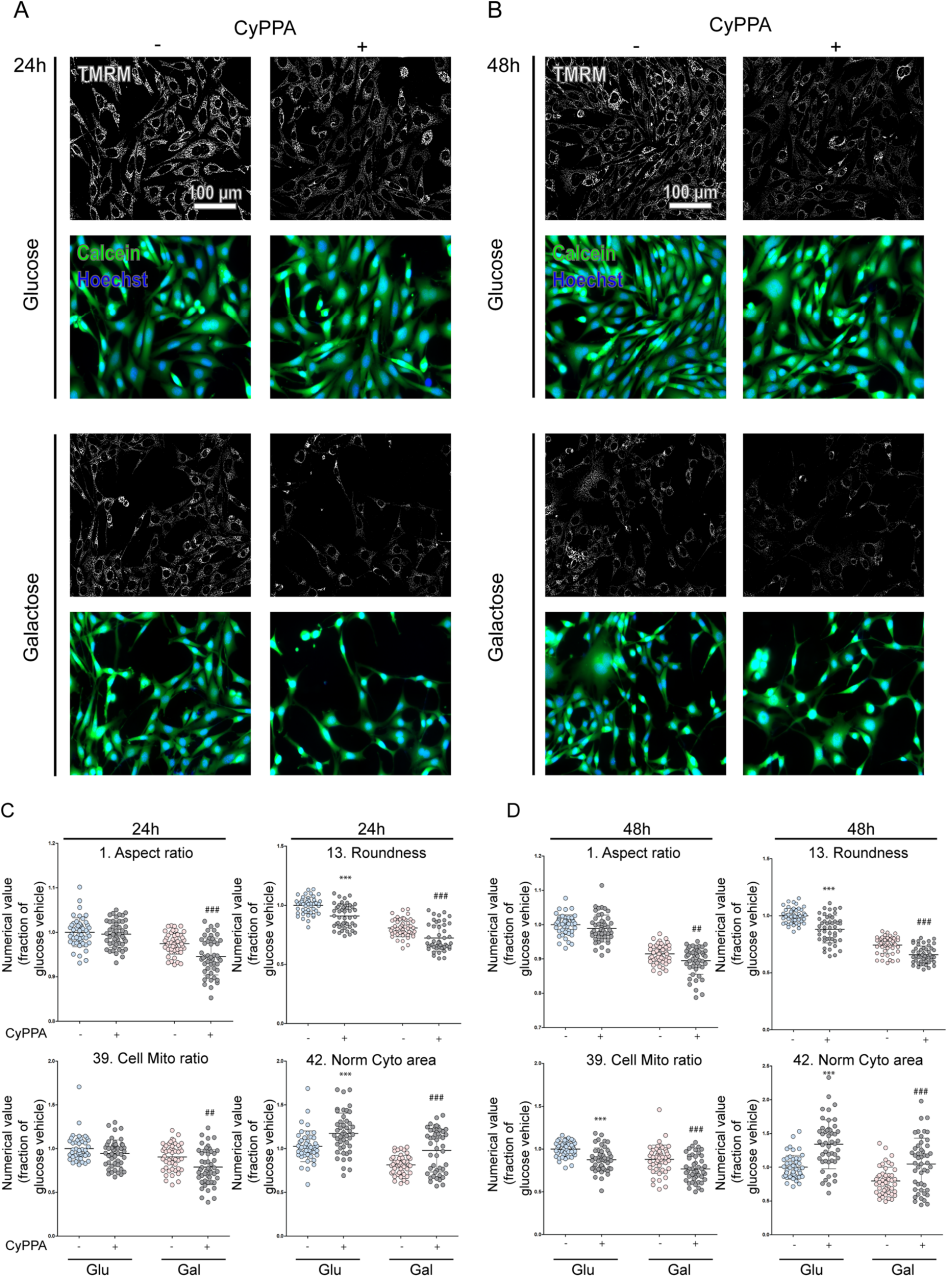
The effects of SK channel activation on mitochondrial morphology. **a**, **b** Representative panel of microscopy images of different conditions and timepoints (TMRM in white, Calcein in green and Hoechst in blue). **c**, **d** Mitochondrial “morphofunction” parameters evaluated by high-resolution microscopy are shown. Aspect ratio, cell mito ratio and roundness are descriptors extracted from TMRM. Integration of cellular and nuclear descriptors allows calculation of various ‘derived descriptors’ of which ‘norm cyto area’ is depicted in the lower right panel. Panels in **c** represent parameters after 24 h of CyPPA (1 µM) treatments, in **d** after 48 h of treatment in glucose (blue dots) or galactose (pink dots). Data are presented as mean ± SD, *n* = 3, ***p < 0.001 versus glucose alone, ^##^*p* < 0.01, ^###^*p* < 0.001 versus galactose only-treated cells. All experiments were independently repeated at least three times.

In order to delineate the effects of CyPPA on mitochondrial function, a mitochondrial “morphofunction” analysis was performed. A previously established protocol using multispectral fluorescence and automated microscopy in fibroblasts^35^ was applied to neuronal HT22 cells. The TMRM-stained HT22 image processing and data extraction (Fig. S2A) was performed according to our previously developed protocol on living primary human skin fibroblasts^35^. A representative panel of microscopy images of the different conditions and timepoints is shown in Fig. 3. A measure of mitochondrial length (depicted as ‘aspect ratio’) and of mitochondrial mass (depicted as ‘cell/mito ratio’), was used as descriptors extracted from TMRM microscopy images (Fig. 3C,D, left panels)^35^. TMRM was used as an indicator of both mitochondrial morphology and MMP. Both parameters were significantly reduced in cells treated with CyPPA in galactose compared to galactose alone following 24 h and 48 h treatment. In glucose medium, no difference was observed in mitochondrial length, while only after 48 h a change in mitochondrial mass was apparent. Mitochondrial ‘roundness’, a combined measure of mitochondrial length and degree of branching, and the average cell size were significantly reduced upon CyPPA treatment in both types of media after 24 h and 48 h (Fig. 3C,D). The average mitochondrial intensity of the TMRM signal (‘density mean’, Fig. S3) was reduced in glucose and galactose medium after CyPPA treatment. This is consistent with HT22 cell treatment with CyPPA resulting in a trend towards a reduction of MMP (Figs. 1E and 2E) measured with a different combination of technology and probe (TMRE/FACS, Figs. 1E and 2E versus TMRM/microscopy presented in Fig. 3). Descriptors of cell viability, ‘casum’ and the number of nuclei (Nn) were not affected by CyPPA treatment in glucose at 24 h (Fig. S3B). Cell numbers quantified on the basis of Nn were slightly reduced by SK channel activation in galactose medium (Fig. S3B). After 48 h, in both media the number of nuclei was reduced (Fig. S3C). Overall, these results suggest that CyPPA alone slightly reduced MMP, while in the presence of a cell death inducer, it can prevent cell death and the pronounced detrimental loss of MMP (Figs. 1E, 2E).

**Fig. 4:**
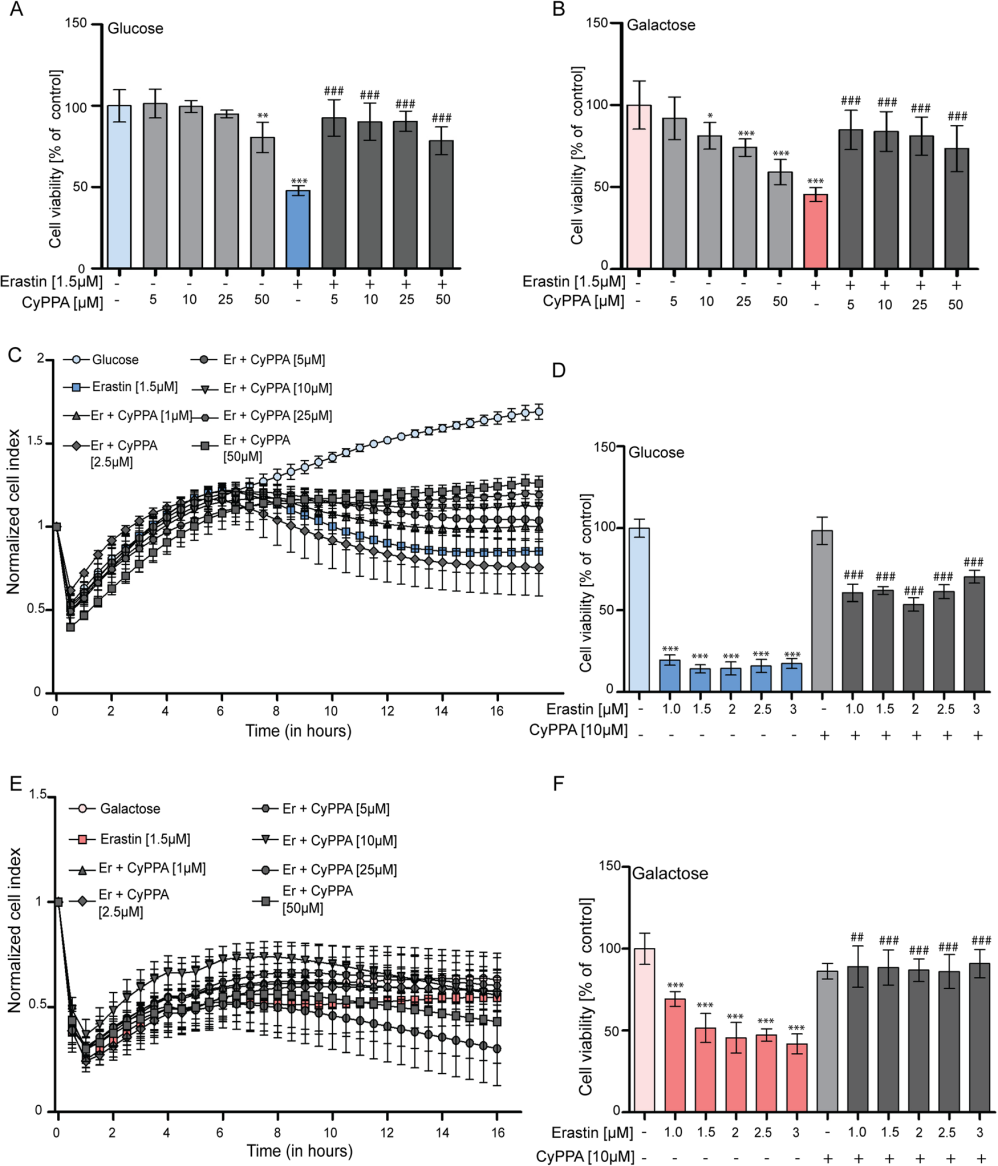
Differential effects in glucose and galactose-based media. **a**, **b** Real-time impedance measurements of HT22 cells treated with CyPPA (5–50 µM, gray curves) in glucose **a** and galactose **b** treated for 42 h. Data are presented as mean ± SD, *n* = 6. **c**, **d** MTT assay of HT22 cells treated with oligomycin, indicated as ‘oligo’ (1, 3, 5 µg/ml, 16 h) in glucose (**c**) or galactose (**d**) in the presence or absence of CyPPA (10 µM, gray bars). **e**, **f** MTT assay in HT22 cells treated with rotenone, indicated as ‘rot’ (1, 2.5, 5, 10, 20 nM, 16 h) in glucose (**e**) or galactose (**f**) in the presence or absence of CyPPA (10 µM). Data are presented as mean ± SD, *n* = 6, *p < 0.05, **p < 0.01, ***p < 0.001, ^#^*p* < 0.05, ^##^*p* < 0.01, ^###^*p* < 0.001, *compared to control, ^#^compared to same rotenone/oligomycin concentration without CyPPA. All experiments were independently repeated at least three times.

Further, the viability of cells grown in glucose or galactose medium in the absence of erastin was assessed by an MTT assay (Fig. S4A,B) and cell impedance measurements (Fig. 4A,C). Our previous studies demonstrated that CyPPA acts by reducing mitochondrial respiration and OXPHOS^19,36^. Although CyPPA (5–10 µM) did not cause any change in cell viability following 24 h of application, the higher concentrations of CyPPA (25–50 µM) slightly reduced cell impedance after 30 h. Glucose replacement with galactose affected cell proliferation and CyPPA reduced cell viability in a dose-dependent manner. Next, cells grown in galactose medium showed reduced cell viability compared to glucose medium in the presence of mitochondrial complex inhibitors (Fig. 4C–F). In addition, CyPPA only increased the effects of oligomycin and rotenone in galactose medium (Fig. 4C–F), in particular upon rotenone-mediated complex I inhibition. Adding galactose as a carbon substrate renders the cells less susceptible to erastin-induced cell death, however, sensitizes them towards oligomycin, rotenone and to CyPPA itself. Taken together, these data suggest that CyPPA-mediated effects on mitochondrial function and cell survival depend on glucose availability as an energy substrate for glycolysis.

### SK channel-mediated increase in glycolytic activity is essential for neuroprotection

Based on our findings, we hypothesized that in glucose medium, CyPPA provided protective effects through a metabolic shift to an increase in glycolytic activity.

**Fig. 5:**
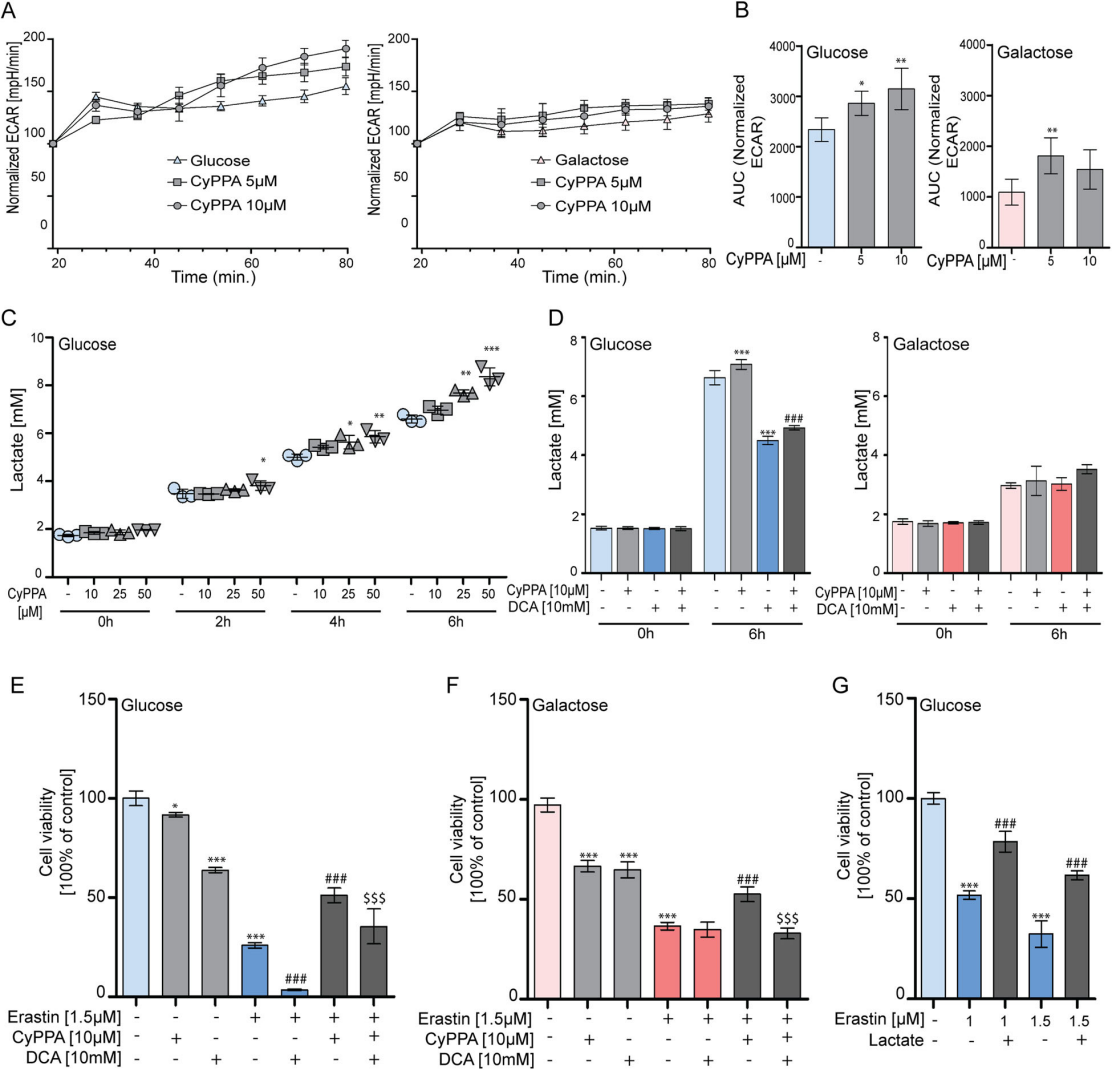
SK channel-mediated increase in glycolytic activity is critical for neuroprotection. **a**, **b** Representative of the normalized extracellular acidification rate (ECAR) in HT22 cells following the application of CyPPA (5 and 10 µM). Left panel depicts cells treated in glucose, right panel treated in galactose. **b** represents area under the curve quantification of the ECAR values presented in panel **a**, with background subtraction. Data are presented as mean ± SD, *n* = 3–6 per condition, *p < 0.05, **p < 0.01 ***p < 0.001 compared to control. **c** Lactate measurement in medium of HT22 cells in glucose after treatment with CyPPA (10, 25, 50 µM) for 0, 2, 4, or 6 h. Data are presented as mean ± SD, *n* = 3 per condition, *p < 0.05, **p < 0.01, ***p < 0.001 CyPPA treatment versus control for the same timepoint **d** Lactate release following treatment with CyPPA (10 µM) in the presence or absence of dichloroacetate (DCA, 10 mM) for 6 h in glucose (left panel) or galactose (right panel) in HT22 cells (12.000 cells/well). Data are presented as mean ± SD, n = 3 per condition, **p < 0.01, ***p < 0.001 CyPPA treatment versus control for the same timepoint, ^###^*p* < 0.001 combination DCA + CyPPA treatment versus CyPPA alone for the same timepoint. **e**, **f** MTT assay in HT22 cells treated with erastin (1.5 µM, 16 h, glucose (**e**), galactose (**f**)) in the presence or absence of CyPPA (10 µM gray bars) and pre-treated for 8 h with DCA (10 mM) and CyPPA. Data are presented as mean ± SD, *n* = 6, ***p < 0.001, ^###^*p* < 0.001, ^$$$^*p* < 0.001, *compared to control, ^#^compared to erastin, ^$^compared to erastin ^+^ CyPPA. **g** MTT assay of HT22 cells pre-treated for 6 h with lactate (150 mM) and challenged with erastin (1 or 1.5 μM) for 16 h. All experiments were independently repeated at least three times.

In order to investigate whether SK channel activation actually affects glycolysis, mitochondrial bioenergetics, in particular the extracellular acidification rate (ECAR) was assessed. Although O_2_ consumption rate (OCR) values did not significantly change in response to short-term CyPPA application (Fig. S5D), there was a fast and significant increase in ECAR following CyPPA application, that lasted at least 1 h (Fig. 5A,B). In galactose-based medium, however, CyPPA treatment had milder effects on glycolysis, with no changes in the glycolytic reserve (Fig. S5A,B), ATP production (Fig. S5C), and glycolytic capacity (Fig. S5E,F).

To further strengthen our findings, we assessed lactate release as another readout of the glycolytic activity. The enzymatic lactate assay showed a dose-and time-dependent increase in lactate levels in response to CyPPA (Fig. 5C), an effect that was clearly limited in cells grown in galactose medium (Fig. 5D). To investigate the molecular mechanisms underlying lactate release in response to CyPPA stimulation, cells were treated with dichloroacetate (DCA) that inhibits pyruvate dehydrogenase kinase (PDK). DCA attenuated CyPPA-induced lactate in glucose-based medium, while it elicited no effects in galactose-based medium (Fig. 5D). DCA alone slightly reduced cell viability and metabolic activity (Fig. S6A,D), this effect being more pronounced in galactose medium, and did not protect against erastin treatment in glucose- and galactose-based medium (Fig. S6B–E). Notably, CyPPA-mediated protection was not completely blocked by co-treatment with DCA indicating that additional mechanisms may be involved beyond the glycolytic increase (Fig. 5E,F, Fig. S6C,F).

To further study whether the CyPPA-mediated-increase in lactate was essential for neuroprotection, cells were pre-treated for 6 h with lactate before the erastin challenge. Concentrations of lactate lower than 150 mM did not exert protective effects (Fig. S6G); however, cell viability assessments revealed that pre-treatment with lactate protected against erastin in a range of 150–200 mM (Fig. 5G). Overall, these findings further support the concept that a metabolic change towards glycolysis plays a key role in the SK channel-mediated protection against erastin-induced ferroptosis.

### SK channel activation reduces the activity of mitochondrial complex I and II

Our previous studies showed that CyPPA reduced mitochondrial respiration^19,36^. Here, we observed that CyPPA is also able to induce an immediate increase in glycolytic activity that might contribute to protection against ferroptosis.

**Fig. 6:**
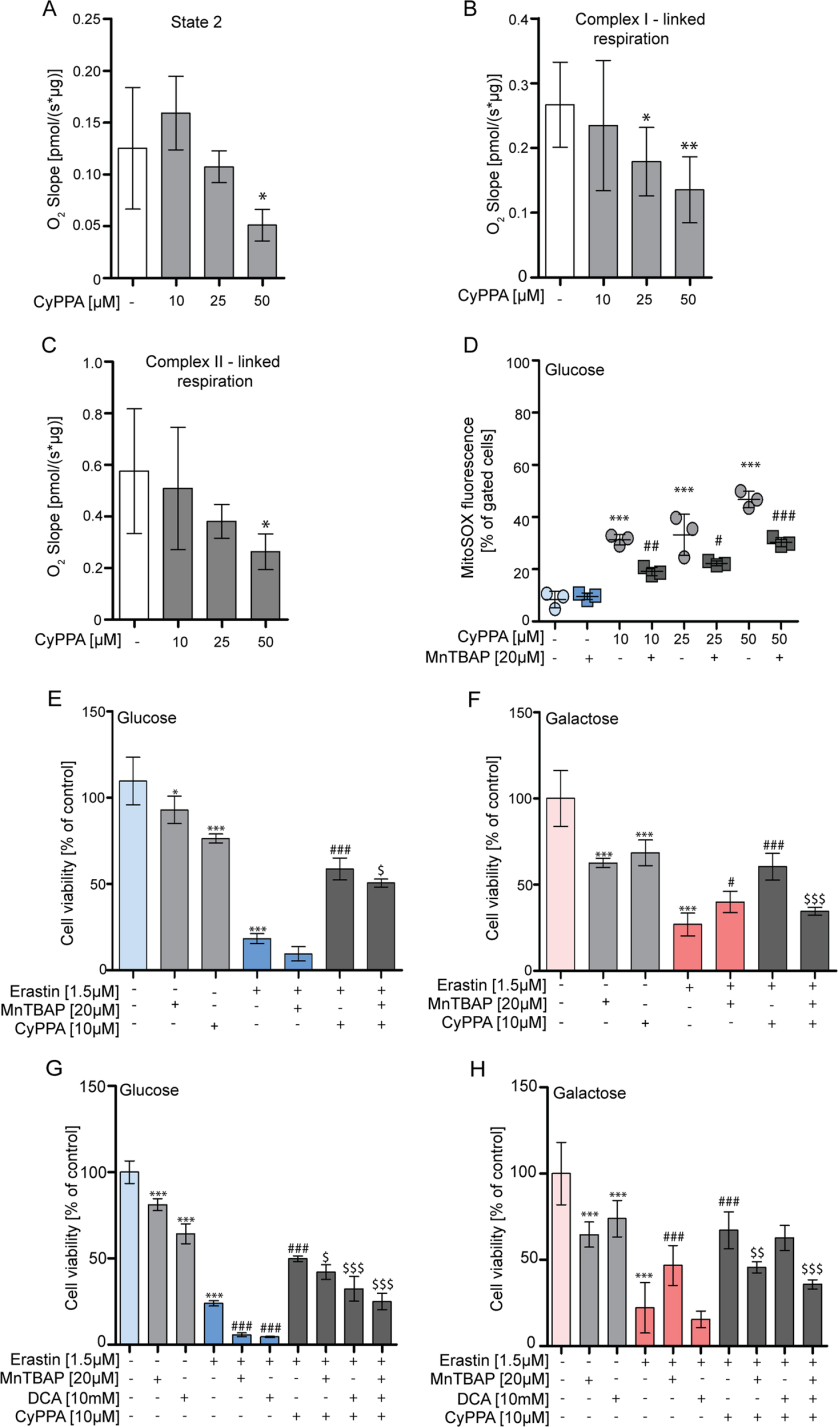
Mitochondrial ROS is essential for neuroprotection. **a**–**c** High-resolution respirometry measurements of the oxygen slope of isolated mitochondria treated for 10 min with CyPPA (10–50 µM). **a** measurements of state 2, basal respiration after addition of pyruvate, malate, glutamate. **b** complex I-linked respiration was determined after addition of ADP. **c** complex II-linked respiration after addition of succinate. Data are presented as mean ± SD, *n* = 3–5 independent experiments per condition. *p < 0.05, **p < 0.01. Statistical significance is assessed by one-way ANOVA Dunnet’s multiple comparisons. **d** Mitochondrial superoxides were measured using MitoSOX after CyPPA (10–50 µM, 24 h) treatment in the presence or absence of MnTBAP (20 µM). Data are presented as mean ± SD, *n* = 3, *p < 0.05, ***p < 0.001 versus untreated control, ^#^*p* < 0.05, ^##^*p* < 0.01 versus CyPPA only-treated cells with the corresponding concentration. **e**–**h** MTT assay of cells challenged with erastin, pre (8 h)- and co-treated with CyPPA (10 µM) in the presence or absence of MnTBAP (20 μM) in glucose (**e**) and galactose (**f**). The combination MnTBAP (20 μM) and DCA (10 mM) is depicted in **g** (glucose) and **h** (galactose). Data are presented as mean ± SD, *n* = 6, *p < 0.05, **p < 0.01, ***p < 0.001, ^##^*p* < 0.01, ^###^*p* < 0.001, ^$^*p* < 0.05, ^$$^*p* < 0.01, ^$$$^*p* < 0.001, *compared to glucose or galactose control, ^#^com*p*ared to erastin, ^$^com*p*ared to erastin and CyPPA. All experiments were independently repeated at least three times.

To study whether the decrease in mitochondrial respiration correlated with a decrease in the activity of mitochondrial complexes, we assessed the direct effect of CyPPA on mitochondrial complex I- and II-dependent respiration. High-resolution respirometry measurements revealed that CyPPA treatment significantly reduced basal respiration and mitochondrial complex I- and II-linked respiration (Fig. 6A–C), whereas lower concentrations exerted milder effects. However, 10 µM CyPPA did reduce mitochondrial respiration after 6 h treatment, as indicated by Seahorse measurements (Fig. S8C). In addition, the maximum uncoupled respiration was reduced in the presence of CyPPA, which was measured after uncoupling the respiratory chain with protonophore FCCP (Fig. S7A). All CyPPA concentrations preserved cell survival in conditions of ferroptosis (Fig. S1A,C).

### SK channel activation induces neuroprotection by mitochondrial preconditioning

Since inhibition of the glycolytic activity only partially reduced the neuroprotection mediated by CyPPA and since opening of the channels slightly decreased complex I and II activity, this prompted us to investigate whether SK channel activation provokes a mitochondrial preconditioning effect. To this end, we treated cells with CyPPA and analyzed mitochondrial superoxide levels and MMP, observing that SK channel activation for 24 h increased mitochondrial ROS levels in a dose-dependent manner (Fig. 6D), and slightly decreased the MMP (Fig. S7B).

Since mitochondrial ROS are pivotal for mitochondrial preconditioning, we next investigated whether the production of mitochondrial ROS was a prerequisite for CyPPA to provide protection against ferroptosis. To this end, cells were treated with CyPPA and manganese(III)-tetrakis(4-Benzoic Acid)porphyrin (MnTBAP), a mitochondria-targeted SOD mimetic and peroxynitrite scavenger^37,38^, prior to and during ferroptosis induction to scavenge mitochondrial ROS induced by CyPPA treatment. Treatment with MnTBAP alone exhibited no effects on cell viability (Fig. S7D,G), and pre-treatment did not protect against erastin (Fig. S7E) in glucose. In galactose medium, pre-treatment with MnTBAP slightly protected against erastin damage, whereas co-treatment did not (Fig. S7H,I). Notably, MnTBAP alone was able to decrease mitochondrial ROS levels in the presence of glucose as well as galactose, and galactose-induced ROS levels were higher compared to glucose (Fig. S7C). This might explain the observed protection against erastin following pre-treatment with MnTBAP in galactose medium (Fig. 6F,H, Fig. S7I). Notably, in the presence of CyPPA, MnTBAP reduced mitochondrial ROS levels and diminished the CyPPA-mediated protection (Fig. 6D–F, Fig. S7F,J). Thus, CyPPA-induced protection is mediated by a mild mitochondrial ROS production that can be attenuated by ROS scavengers.

**Fig. 7:**
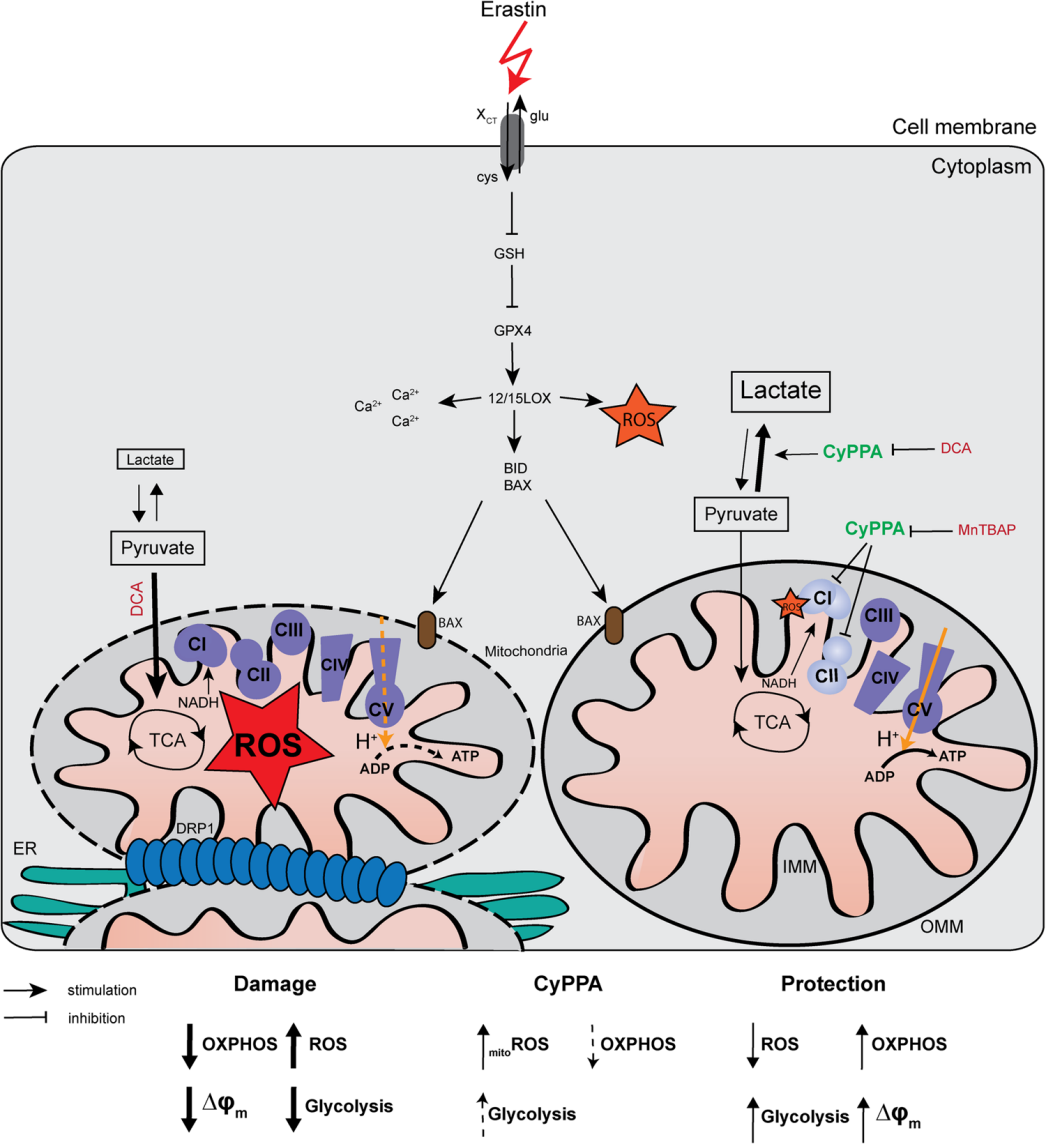
Activation of SK channels prevents ferroptotic cell death. Erastin blocks the Cys/Glu antiporter (XCT) in the plasma membrane leading to loss of glutathione (GSH) and glutathione peroxidase 4 (GPX4) activity. This enhances the oxidation of lipids by 12/15-lipoxygenases (12/15LOX), increasing ROS levels, promoting mitochondrial Ca2+ influx and increased ROS levels. Furthermore, ER wraps around mitochondria and initiates mitochondrial fragmentation, concomitant with the recruitment of dynamin-related protein 1 (DRP1) at the mitochondria to trigger mitochondrial fission. Activation of SK channels by CyPPA slightly decreases mitochondrial complex I activity and induces a mild increase in mitochondrial ROS levels. This leads to preconditioning effects, resulting in attenuation of ROS levels in conditions of oxidative stress. However, in the presence of MnTBAP, ROS are scavenged and this reduces the CyPPA-induced protection. In addition, CyPPA increases lactate production, which contributes to the observed protection. DCA promotes pyruvate to enter the citric cycle and facilitate more OXPHOS, thereby attenuating the CyPPA effect on glycolysis. Thus, opening of SK channels contribute to cell survival by increasing glycolytic activity and preventing mitochondrial damage.

Assuming that CyPPA enhances glycolysis while reducing mitochondrial respiration in glucose conditions, we next assessed whether blocking both mitochondrial ROS and glycolysis, would further prevent neuroprotection during ferroptosis. To test this, we combined CyPPA with a treatment of DCA and MnTBAP in conditions of ferroptosis (Fig. 6G,H) and detected that the protection was completely abolished. These results strongly suggest that both glycolysis and mitochondrial ROS levels are required for the observed protective effects of SK channel activation against ferroptosis (Fig. 7).

### SK channel activation protects against paraquat and supports longevity in *C. elegans*

**Fig. 8:**
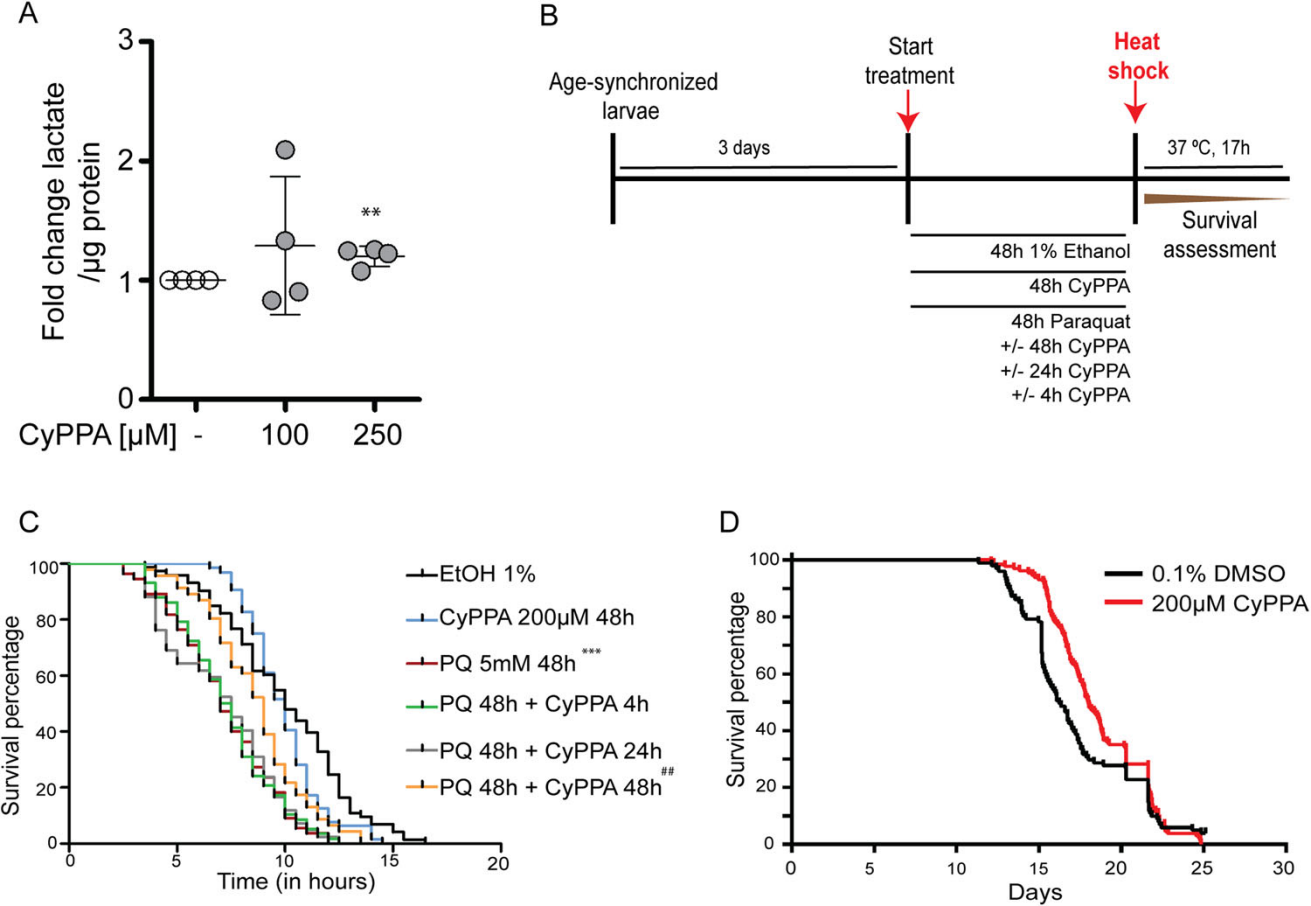
SK channel activation preserves the viability of *C. elegans* in conditions of mitochondrial stress. **a** Lactate measurements in *C. elegans* following four days of CyPPA treatment (100, 250 µM). Data are presented as mean ± SD, **p < 0.01, **b** Schematic representation of the heat-stress experiment. Young adult nematodes were exposed to CyPPA (final concentration 200 µM), paraquat (final concentration 5 mM) and a combination of the two with the same concentrations, with CyPPA treatment for different timepoints. Finally, heat-stress at 37°C is induced to determine lifespan over 17 h **c**. In the presence of paraquat exposure, CyPPA treatment for 48 h resulted in a higher survival rate compared to 4 h or 24 h exposure. Paraquat alone decreased the survival rate (***p < 0.001) and CyPPA (48 h) was able to reverse this effect significantly ^##^*p* < 0.01. **d** Survival curves of wild-type worms treated with 200 µM CyPPA or 0.1% DMSO as control. The experiment was ended before all the control worms had died. CyPPA slightly but significantly (*p* < 0.001) increased the mean lifespan (18.6 ± 3.2 days vs 16.9 ± 2.9 days for the control). Sample sizes are 131 and 101 for 200 µM CyPPA or 0.1% DMSO, respectively.

In order to assess the effects of CyPPA in vivo, we measured lactate in the nematode *C. elegans* growing on CyPPA. Synchronized N2 nematodes were grown on OP50 ± CyPPA and lactate was measured after four days. Lactate levels were increased following treatment with 250 µM CyPPA compared to DMSO-treated nematodes, while 100 µM CyPPA did not affect lactate levels (Fig. 8A). These results indicated that CyPPA was able to increase lactate levels both in vitro in neuronal HT22 cells as well as at a systemic level *C. elegans* in vivo.

Next, SK channel effects in *C. elegans* were studied in a heat-stress survival assay following paraquat exposure. The heat-stress survival assay is an established method to study lifespan over a short period of up to 17 h for drug screening purposes, with predictive potential for the long term lifespan analysis over three weeks^39^. Paraquat induces oxidative stress in nematodes, and indeed it decreased the survival rate of the nematodes. As illustrated in the scheme Fig. 8B, we tested the effects of SK channel activation on paraquat-dependent toxicity by exposing the worms to CyPPA for different timepoints following the initiation of the paraquat insult. CyPPA treatment for 4 h or 24 h following the insult was not sufficient to mediate full protection against paraquat-induced death. Importantly, CyPPA treatment for 48 h was able to attenuate the paraquat toxicity significantly, resulting in a higher survival rate (Fig. 8C). Paraquat induced cell death in HT22 cells as well, from concentrations starting at 350 μM. Importantly, CyPPA preserved cell viability for concentrations of paraquat up to 500 μM (Fig. S8A,B).

To determine the effect of CyPPA on the lifespan of *C. elegans*, we grew wild-type worms for two generations on NGM plates containing 200 µM CyPPA and monitored their time of death using the Lifespan Machine^40^. We found that CyPPA slightly extended the mean lifespan of the wild-type animals. No effect on the maximum lifespan was observed (Fig. 8D). Taken together, we confirmed that CyPPA targets metabolic programs also in vivo, thereby conferring protection against oxidative stress in nematodes.

## Discussion

In the present study, we show that SK channel activation protects against erastin-induced ferroptosis by mechanisms involving increased initial glycolysis, and a mild induction of mitochondrial ROS formation. In this study we delineated consequences of the ability of cells to use glycolysis as an energy source in conditions of SK channel activation. We show that CyPPA mediated protection against ferroptosis, as shown by reduced cell death, and attenuated mitochondrial ROS levels in medium containing glucose. We suggest that a moderate inhibition of mitochondrial respiration, complex I activities, OXPHOS and the concomitant metabolic shifts are prerequisites for CyPPA-mediated protection. However, this inhibition also results in enhanced vulnerability to cell death when cells were grown in the galactose medium where mitochondrial respiration is the dominating process for energy supply. This prompted us to study the effects of SK channel activation on glycolytic activity. Our results revealed that SK channel activation mediates a fast increase in glycolytic activity and an increase in lactate levels. By increasing the glycolytic capacity, cells can adapt to maintain energy production in conditions of deficient mitochondrial respiration. The metabolic switch to aerobic glycolysis, characterized by increased lactate levels, is a common phenotype of cancer cells^41–44^. This phenomenon has also been identified in neurodegenerative diseases such as AD^45^. For example, increased activity of the glycolytic enzymes pyruvate kinase and lactate dehydrogenase A (LDHA) were elevated in the brain cortex of AD patients^46^. Increased levels of PDK1, and concomitantly increased activity of LDHA led to enhanced aerobic glycolysis, which protected against Aß toxicity in various neuronal lines^47,48^. DCA is commonly used for its property to inhibit PDK activity, leading to increased entry of pyruvate to the TCA. Thus, cells exposed to DCA undergo more OXPHOS at the expense of glycolysis. Treatment with this compound was shown to reverse the glycolytic phenotype in cerebellar granule cells undergoing apoptosis^49^. DCA allows glycolysis to take place, but it constrains the aerobic glycolytic pathway observed in many cancer cells and effectively kills them with limited cytotoxicity on healthy cells^50–53^. In our study, when HT22 cells were co-treated with DCA, both CyPPA-induced lactate production and the protection against ferroptosis were significantly decreased. In addition, pre- and co-treatment with lactate during erastin stimulation prevented cell death. Even though the increase of lactate release in response to CyPPA confirms that glycolysis was enhanced, whether the lactate may contribute to corresponding protection remains to be investigated. Although high lactate levels result in acidosis in certain forms of mitochondrial diseases, as well as in stroke, recent data indicate that lactate, the end-product of glycolysis, may act as an alternative energy substrate and could promote neuroprotection in models of ischemic cell death^54^. Extracellular application of lactate induced protection against erastin toxicity, although the protective concentrations of lactate were 10 times higher than those resulting from the cellular lactate release. Therefore, the expression profile of lactate transporters, such as proton-linked-monocarboxylate-transporters (MCTs) and Na^+^-coupled-electrogenic-transporters in HT22 cells might provide evidence for these effects. Indeed, administration of pyruvate after oxygen-glucose deprivation enhanced the expression of MCT2 and mediated neuroprotection in HT22 cells^55^. The neuronal MCT2 transporter may serve to maximize the available energy sources to the damaged cells. Furthermore, transient and/or moderate ROS increase can initiate signaling pathways involved in the stimulation of glucose uptake, as a result of a cellular adaptation to metabolic stress conditions^56^. Overall, these data support the importance of glycolysis in SK channel-mediated neuroprotection in conditions of ferroptosis.

In our recent studies, SK channel activation was associated with moderately increased mitochondrial ROS and attenuated mitochondrial respiration, suggesting a potential involvement in preconditioning pathways^19,36,60^. Partial inhibition of mitochondrial complex I by low concentrations of rotenone provided protection in HT22 cells against glutamate-induced oxidative toxicity^57^. Our current results demonstrate that CyPPA decreased the activity of mitochondrial complex I at higher concentrations than 25 μM on isolated mitochondria, while it already mediated cytoprotective effects at lower concentrations. These acute effects on mitochondrial complex activities were measured on crude mitochondria, whereas OCR measurements revealed a decrease in OXPHOS in whole cells after 6 h of CyPPA treatment, suggesting that the initial increase in glycolytic activity and the late moderate decrease in mitochondrial respiration are crucial for CyPPA protection. In addition, opening of SK channels alone resulted in a slight decrease in MMP as detected by the FACS and mitochondrial morphofunctional analysis. Slight decreases in MMP have been linked before to preconditioning-dependent neuroprotective pathways^58,59^, whereas pronounced loss of the MMP results in mitochondrial dysfunction and cell death^60^. Furthermore, when mitochondrial ROS initiated by CyPPA were scavenged by the antioxidant MnTBAP, the CyPPA-mediated protection was attenuated. Several studies showed that MnTBAP is neuroprotective against oxidative stress^61,62^, while it may block the protective effect of large conductance calcium-activated K^+^ channel (BK) channels in models of ischemia-reperfusion injury^63^, which is in line with our current observations. Interestingly, when combining DCA and MnTBAP, the protection mediated by CyPPA was even further reduced, suggesting that both glycolysis and mitochondrial ROS are essential in SK channel-mediated neuroprotection.

More evidence for SK channel-mediated protection against oxidative stress derives from our in vivo data. We demonstrated in *C. elegans* that SK channel activation mediated an increase in lactate production and an improved survival after heat-stress induction. Heat-stress is an established model mimicking neuronal decline^64^. These findings in vivo correspond well with our observations in HT22 cells in vitro, where CyPPA also provided protection against paraquat. Moreover, we found that CyPPA extended the mean lifespan of *C. elegans*. Previous studies established that interfering with mitochondrial function can significantly extend lifespan in *C. elegans*^28,65^. Notably, a modest decrease in GSH levels in young adult worms was shown to mediate stress resistance and promote lifespan^66^. It has been suggested that an increase in mitochondrial ROS serves as a preconditioning effect, which involves activation of antioxidant defense mechanisms sustaining cellular resilience^67,68^.

In conclusion, our study shows that the activation of SK channels provides protection against cell death by initiating a metabolic shift towards glycolysis. This metabolic shift involves a decrease of mitochondrial complex activity and induction of mitochondrial preconditioning that renders the cells resistant to oxidative damage.

## Materials and methods

### Cell culture

HT22 cells were cultured in Dulbecco’s modified Eagle Medium glucose (DMEM; Gibco, Thermo Fisher Scientific, the Netherlands) enriched with 10% fetal bovine serum (FBS; GE Healthcare Life Sciences, the Netherlands), 100 U/mL penicillin/streptomycin, and 1 mM sodium pyruvate (Gibco, Thermo Fisher Scientific, the Netherlands) at 37 °C and 5% CO_2_. HT22 cells were kindly provided by Prof. Carsten Culmsee (University of Marburg, Germany) and the passage number used in this study ranged between 270 and 350. Cells were regularly tested for Mycoplasma contamination and the study was performed with Mycoplasma free cells. The treatment media were prepared supplementing DMEM, no glucose, no glutamine, no phenol red with: 1 mM sodium pyruvate, 1mM L-glutamine (not in the case of galactose medium), 10% FBS, 100U/ml penicillin/streptomycin, 0.05 mM Phenol Red (Sigma-Aldrich), and 25 mM glucose (Sigma-Aldrich) for glucose assay medium or 25 mM galactose (Sigma-Aldrich) for galactose-based medium. HT22 cells were challenged with erastin (Tocris) or various mitochondrial complex inhibitors, including rotenone (complex I) (Sigma-Aldrich) and oligomycin (complex V) (Sigma-Aldrich) for 16 h, or with paraquat (Sigma-Aldrich) for 24 h. We have obtained similar results when pyruvate was present or absent in the galactose media. SK2 channels were pharmacologically activated with the selective SK2/SK3 channel activator N-Cyclohexyl-N-[2- (3,5-dimethyl-pyrazol-1-yl)-6-methyl-4-pyrimidinamine (CyPPA). In attempt to reduce the CyPPA-mediated protection against erastin, the compounds MnTBAP and dichloroacetate (Sigma-Aldrich) were used in a pre-treatment period of 8 h and in the presence of erastin. Sodium L-lactate (Merck Millipore) was used for cell culture treatments for 6 h before and during erastin stimulation.

### Cell viability

Cells were seeded with a density of 10.000 cells/well 24 h before treatment. Cell viability was determined based on the metabolic activity using the 3-(4,5-dimethylthiazol-2-yl)-2,5-diphenyltetrazolium bromide (MTT) assay at a final concentration of 0.5 g/L by incubation for 1 h at 37 °C, followed by removal of the MTT (Sigma-Aldrich, Zwijndrecht, the Netherlands) and at least 1 h incubation at −20 °C. After dissolution of the resulting formazan in DMSO, the absorbance of each well was determined with the Synergy H1 Multi-Mode reader (Biotek, LA, USA) at 570 nm and at 630 nm. Alternatively, cell viability was monitored in real-time with cell impedance measurements, using the xCELLigence system (Roche Diagnostics, Penzberg, Germany). Cell impedance was normalized to the time of treatment (normalized cell index), which is defined as the starting point (*t* = 0 h) of the experiment.

### Mitochondrial morphofunction

#### Assay medium preparation, cell seeding and CyPPA treatment for mitochondrial morphofunctional analysis

The culture medium was prepared supplementing DMEM, high glucose (#41965039; Thermo Fisher) with: 10% (v/v) Fetal Bovine Serum (FBS; #758093; Greiner Bio-One, Kremsmünster, Austria), 100 IU/ml penicillin/streptomycin (#30-002-CI; Corning).

The assay medium was prepared supplementing DMEM, no glucose, no glutamine, no phenol red (#A1443001; Thermo Fisher) with: 1 mM sodium pyruvate (#11360070, Life Technologies, Carlsbad, CA, USA), 1 mM L-glutamine (GlutaMAX®; #35050061; Thermo Fisher), 10% (v/v) Fetal Bovine Serum (FBS; #758093; Greiner Bio-One, Kremsmünster, Austria), 10 mM HEPES (#15630080; Thermo Fisher), 100U/ml penicillin/streptomycin (#30-002-CI; Corning), 0.05 mM Phenol Red (#3532; Sigma-Aldrich), and 25.5 mM glucose (#G8270; Sigma-Aldrich, St. Louis, MO, USA) for glucose assay medium or 25.5 mM galactose (#G0750, Sigma-Aldrich) for galactose assay medium. Glucose and galactose assay media were stored at 4 °C in the dark for a maximum of one month. The SK channel activator CyPPA was freshly dissolved in DMSO and diluted in assay medium to a final concentration of 1 µM before every experiment.

Cells were seeded in culture medium in black 96-well plates (#655090, Greiner Bio-one) at a density of 4000 cells/well, and incubated overnight in a humidified atmosphere (95% air, 5% CO_2_) at 37 °C. The next day, culture medium was removed and glucose or galactose assay medium was added. Then, the cells were cultured in these media for 24 h and 48 h when mitochondrial morphofunctional analysis was performed.

#### Mitochondrial morphofunctional analysis

Fluorescence microscopy images were acquired from 96-well plates (#655090, Greiner Bio-One) using a BD Pathway 855® High-Content Bioimager (Becton Dickinson, Franklin Lakes, NJ, USA) using a protocol previously described^35^. Minor adjustments were introduced for HT22 cells related to the microscope exposure time during image acquisition and the threshold to yield a binary (BIN) image during image processing (Fig. S2A). An optimal exposure time of 0.2 s and a threshold value of 60 gray values were empirically determined. All other settings were as previously described. In brief, cells were co-stained with three fluorescent membrane-permeable chemical reporters: TMRM (Tetramethyl rhodamine methyl ester, #T668, Invitrogen), Calcein-AM (#65-0853-39; Thermo Fisher), and Hoechst 33258 (#94406, Invitrogen). TMRM is a cation that accumulates in the mitochondrial matrix according to the magnitude of the MMP (depicted in white) Calcein-AM passively accumulates in the cytosol and upon esterase-mediated cleavage of its AM (acetoxymethyl) ester tail it becomes green fluorescent. Hoechst 33258 binds to the AT base pairs in the minor groove of double-stranded DNA greatly increasing its blue fluorescence signal. Combination of cellular and nuclear descriptors allows for calculation of various ‘derived descriptors’ of which ‘norm cyto area’ reflects a measure of average cell size.

### Seahorse XF analysis

HT22 cells were seeded in Seahorse XF 96-well plates (Seahorse Biosystems). For the experiment with different concentrations of CyPPA in either glucose-based or galactose-based medium, the medium was removed before the measurement and replaced by 180 μL assay medium containing 2mM L-glutamine, 1 mM pyruvate, 1 g/L BSA and either 25 mM glucose or 25 mM galactose (pH 7.35), for 2 h at 37 °C. The Seahorse XF Biosystem was used to analyze oxygen consumption rate (OCR) and extracellular acidification rate (ECAR). Three baseline measurements (4x min mix, 0 min delay, 4 min measure = 4/0/4) were recorded followed by injection of CyPPA (0–10 μM) or medium and measurement for 2 h (4/0/4). Mitochondrial metabolism was assessed by injection of 3 µM oligomycin (4/0/4), 50 µM dinitrophenol (DNP) (4/0/4), and 50 mM 2-deoxy-D-glucose (2-DG) (4/0/4). After injection of each compound, OCR and ECAR were determined. Glycolytic reserve was calculated by subtracting the first two ECAR values after oligomycin injection. Subtracting the ECAR values after 2-DG injection from the maximum ECAR values in response to oligomycin application provided the values of the glycolytic capacity.

In addition, HT22 cells were treated with 10 μM CyPPA for 6 h, followed by removal of the medium and replacement by 180 μL assay medium containing 2mM L-glutamine, 1 mM pyruvate and 25 mM glucose (pH 7.35). The plate was incubated for 1 h at 37 °C without CO_2_. Three baseline measurements (3x min mix, 0 min delay, 3 min measure = 3/0/3) were recorded, followed by assessment of mitochondrial metabolism by injection of 4 µM oligomycin (3/0/3), 50 µM DNP (3/0/3), 150 nM rotenone and 1 µM antimycin A, and 50 mM 2-deoxy-D-glucose (2-DG) (3/0/3).

### Lactate measurement in medium from HT22 cells

Cells were seeded in a density of 12.000 cells/well, 24 h later followed by treatments. Cells were treated with different CyPPA concentrations for 6 h and lactate release was monitored every 2 h. Medium from HT22 cells was collected and diluted 10 times in demineralized water. A calibration curve of eight lactate standards ranging from 0 to 1.2 mM was prepared for quantification purposes. Subsequently, lactate was measured in a 96-well plate using 20 µl medium sample or lactate standard mixed with 225 µl reaction mixture (0.44 M Glycine / 0.38 M Hydrazine [pH 9.0], 2.8 mM NAD) and 5 units L-lactic dehydrogenase (EC 1.1.1.27) followed by absorbance determination at 340 nm using the Synergy^TM^ H4. All chemicals were purchased from Merck Millipore. Background absorbance of the blank control (0.0 mM lactate standard) was subtracted from all sample readings and medium samples were corrected for dilution. Medium lactate concentrations were determined based on linear regression of the standard curve.

### High-resolution respirometry in mitochondria from HT22 cells

Mitochondrial respiration was analyzed by high-resolution respirometry with the oxygraph O2K (Oroboros systems, Innsbruck, Austria). HT22 cells (5–9 × 10^7^) were collected, and mitochondria were extracted using a pump-controlled system as described^69^. In brief, dissolved cell pellets in isolation buffer (250 mM sucrose, 20 mM HEPES, 3 mM EDTA, pH 7.5) were gently passed through the Balch homogenizer with 2 ml glass syringes fitted with a metal lock for three times^70^. To break open and homogenize the HT22 cells a 10 µm ball clearance was used. The homogenate was centrifuged at 800 × *g* for 10 min at 4 °C to discard debris. The mitochondria containing supernatant was collected and re-centrifuged at 9000 × *g* for 10 min at 4 °C. The crude mitochondria- containing pellet was resuspended in 200 µl of mitochondrial respiration medium MirO5 buffer (0,5 mM EGTA, 3 mM MgCl_2_, 60mM K-lactobionate, 20 mM taurine, 10 mM KH_2_PO_4_, 20 mM HEPES, 110 mM sucrose, 1 g/L BSA, pH 7,4) developed by Oroboros^71^. The mitochondrial respiration of an aliquot of 200 μg crude mitochondria was monitored under continuous stirring at 750 rpm at 37 °C in 1 ml MiR05 buffer after treatment for 10 min with vehicle (ethanol) or CyPPA (10–50 μM). For each measurement 200 µg of treated mitochondria was added into the 2 ml of air-saturated MirO5 containing electrode chamber.

Oxygen flux per tissue mass (pmol O_2_ s^−1^ mg^−1^) was recorded in real-time using the DatLab software. Non-phosphorylating respiration was induced by adding the complex I-linked substrates pyruvate (5 mM) glutamate (10 mM) and malate (2 mM). The OXPHOS-capacity of complex I-linked activity was measured after addition of a saturating concentration of ADP (2.5 mM). Succinate was applied (10 mM) to determine OXPHOS-capacity of complex II-linked activity. The maximum respiration was monitored by stepwise titration of the protonophore carbonyl cyanide 4-(trifluoromethoxy) phenylhydrazone (FCCP) in 1 μM steps. The oxygen concentration and the first derivative of the oxygen concentration, reported as the oxygen consumption given in the O_2_ slope (pmol × [ml/s]) of crude isolated mitochondria were recorded at 2 s intervals using instrumental background correction after calibration of the polarographic oxygen sensors. The oxygen consumption slope rates were normalized per µg protein.

### Cell death assay

For AnnexinV/PI double staining, the cells were cultured in a 24-well plate format and harvested by using Trypsin/EDTA after treatment. Cells were incubated in binding buffer containing AnnexinV-FITC and propidium iodide (PI) (Fisher Scientific, Landsmeer, Netherlands) at room temperature for 5 min. Flow cytometric analysis was performed with excitation at 488 nm and emission at 525/530 nm for AnnexinV and at 690/50 nm for PI using the CytoFLEX (Beckman Coulter, Woerden, the Netherlands). Dead cells were quantified from AnnexinV, PI and AnnexinV+PI-positive cells. Data were collected from 15000 cells per condition.

### Measurement of mitochondrial superoxide production

Formation of mitochondrial superoxides was determined with the MitoSOX dye (Fisher Scientific, Landsmeer, Netherlands). The MitoSOX dye has been proven to be located within mitochondria in HT22 cells, which was not influenced by oxidative stress stimuli^36^ Cells were incubated with 2.5 µM MitoSOX dye for 30 min at 37 °C and then harvested. Fluorescence was excited at 488 nm and detected at 690/50 nm using CytoFLEX (Beckman Coulter, Woerden, the Netherlands). Data were recorded from 1.5 × 10^4^ cells in triplicate per condition.

### Measurement of the mitochondrial membrane potential (MMP)

Loss of the MMP was assessed with tetramethyl rhodamine-ethyl ester (TMRE; Fisher Scientific, Landsmeer, Netherlands) dye. The TMRE dye has been proven to be located within mitochondria in HT22 cells, which was not influenced by oxidative stress stimuli^36^. Cells were collected and incubated 30 min with 0.2 µM TMRE at 37 °C. TMRE fluorescence was excited at 488 nm and detected at 690/50 nm using CytoFLEX (Beckman Coulter, Woerden, the Netherlands). Data were recorded from 1.5 × 10^4^ cells in triplicate per condition.

### Lactate measurement in *C. elegans*

The *C. elegans* strain N2 was grown on NGM plates and on OP50 bacteria as a food source. For the lactate measurement, the nematodes were synchronized by bleaching with NaOH/NaClO and grown on NGM plates containing OP50 and either DMSO, 100 µM CyPPA or 250 µM CyPPA for 4 days. To determine lactate levels, the lactate assay kit was used (Sigma-Aldrich, MAK064). Briefly, worms were collected and washed to remove OP50. The worm pellet was snap-frozen, resuspended in lactate assay buffer and sonicated to break the cuticle. After centrifugation at 13000 rpm for 10 min, the supernatant was applied to a 10 kDa MWCO filter and centrifuged for 60 min at 13000 rpm to remove insoluble material. A standard curve was prepared and 50 µL of the supernatant was used for the measurement. Fluorescence intensities (ex535nm/em587nm) were measured using a Tecan Infinite M Plex platereader (Tecan Group Ltd, Switzerland). A Bradford (Sigma-Aldrich, Germany) assay was performed (595 nm) to determine the protein content of the sample. Four independent experiments were performed.

### *C. elegans* heat-stress survival assay

#### *C. elegans* and bacterial strains

*C. elegans* strains were maintained on nematode growth medium (NGM) agar plates seeded with E. coli OP50 at 20 °C according to standard protocols^72^. *C. elegans* wild-type (N2) was obtained from the Caenorhabditis Genetics Center (University of Minnesota, MN, US). For all experiments, synchronous populations were used that were obtained by bleaching^73^.

#### Heat-stress survival assay at 37 °C

Synchronous nematodes were raised in liquid culture using NGM liquid and packed E. coli OP50 according to Stiernagle^73^. The experiments have 4–5 biological repeats and only one technical replicate each time. Carbenicillin was added to the NGM liquid in order to limit further contamination of the growth medium with other bacteria or fungi. A volume of 44 μl of NGM liquid was dispensed in each well of a 96-well microplate (Greiner, Bio-One, Frickenhausen, Germany), to which 10 μl M9 buffer containing 10 synchronized L1 larvae was added. L1 larvae were maintained shaking at 20 °C and reached the young adult stage within 3 days. To young adult nematodes a volume of 6 μl effector was added. Effectors were EtOH:Tween with a final concentration of 1%, CyPPA with a final concentration of 200 μM, paraquat with a final concentration of 5 mM and a combination of the two with the same concentrations. Time till death of the nematodes was determined 48 h later using a microplate thermotolerance assay as described^74^. In brief, nematodes were washed off the wells with M9-buffer into 15 ml tubes followed by additional three washing steps. In each well of a black 384-well low-volume microtiter plate (Greiner Bio-One, Frickenhausen, Germany) 6.5 μl M9-buffer/Tween® 20 (1% v/v) solution was added. Subsequently, one nematode was dispensed in 1 μl M9 buffer under a stereomicroscope (Breukhoven Microscope Systems) into each well and mixed with 7.5 μl SYTOX green (final concentration 1 μM; Life Technologies, Karlsruhe, Germany). To prevent water evaporation the plates were sealed with Rotilab sealing film and covered with a lid (Greiner Bio-One, Frickenhausen, Germany). Heat shock (37 °C) was induced and fluorescence was measured with a ClarioStar Platereader (BMG) every 30 min over the course of 17 h. To detect SYTOX green fluorescence, excitation wavelength was set to 485 nm and emission was measured at 538 nm.

### Lifespan *C. elegans*

CyPPA was dissolved in DMSO and added to the NGM plates to a final concentration of 200 µM. Sample sizes are 131 worms (5 technical repeats) and 101 worms (four technical repeats) for 200 µM CyPPA or 0.1% DMSO, respectively. All the data comes from one biological repeat. 5-fluorodeoxyuridine (FUdR; Sigma F0503) was dissolved in water and added to the NGM to a final concentration of 50 µM. Nystatin suspension (Sigma N1638) was added to the NGM to a final concentration of 100 U/mL. Lifespan analysis was carried out using the Lifespan Machine, essentially as described^40^. Worms were first grown for one full generation on NGM plates containing 200 µM CyPPA. The progeny was transferred to plates containing 200 µM CyPPA and 5-fluorodeoxyuridine (FUdR; Sigma F0503) at the young adult stage. Two days after being transferred to plates containing FUDR, worms were transferred to the plates to be used with the lifespan machine, which were made with the modified NGM as described. These plates also contained 200 µM CyPPA, 50 µM FUDR and 100 U/mL Nystatin. At each step, control plates contained 0.1% DMSO instead of 200 µM CyPPA. 30 worms were transferred to each lifespan machine plate and then placed on the lifespan machine. Each plate was scanned once per hour until all worms appeared to have ceased moving. Time of death was determined using the automated worm detection and movement analysis software^40^ and then validated manually.

### Statistics

Statistical significance was assessed using one-way analysis of variance (ANOVA) followed by Tukey’s comparison (GraphPad Prism 5.0) unless otherwise stated. For high-resolution respirometry experiments, statistics were assessed with ANOVA, followed by Dunnet’s comparison. For the xCELLigence real-time impedance measurements, MTT assays, and Seahorse extracellular flux analysis at least three independent experiments with six technical replicates for each experiment were performed. For FACS measurements and high-resolution respirometry, at least three independent experiments with three technical replicates for each experiment were performed. Representative graphs are shown for the FACS, MTT, lactate, Seahorse, and xCELLigence measurements. For the mitochondrial morphoanalysis the image processing, quantification, and data extraction were performed using Image Pro Plus® software (Media Cybernetics, Rockville, MD, USA) and MATLAB® scripts (Mathworks, Natick, MA., USA), as previously described^35^. Data analysis was performed using Graphpad PRISM® software (Graphpad Software, San Diego, CA, USA). Three independent experiments with sixteen technical replicates each experiment, for a total of 48 replicates per conditions, were performed. For the heat-stress survival experiments, curves with an unreadable timepoint of SYTOX green fluorescence signal increase were excluded. Otherwise all readable outputs were included. In these studies, the readout of signal was automated and all groups were treated in the same way, that no further randomization was necessary. In addition, the blinding was not necessary since definite timepoints of signal increase were given. For the heat-stress survival experiments, statistical analyses were performed with GraphPad Prism 7.0 software (GraphPad, La Jolla, CA, USA). Kaplan–Meier survival curves are shown for survival experiments. Mean lifespans and *C. elegans* lactate release were compared using the Student’s *t*-test (GraphPad Prism 7.03). For the lifespan experiments, animals were randomly assigned to the control and experiment groups. *C. elegans* deaths were automatically determined by the analysis software and those that could not be recognized were automatically excluded by the recognition software. For all small sample sizes (<5), individual data points are plotted. *p*-values indicating statistically significant differences between the mean values are defined as follows: *p < 0.05, **p < 0.01, and ***p < 0.001. Data are presented as mean ± standard deviation (SD).

## Supplementary information

Supplementary figure legends

S1

S2

S3

S4

S5

S6

S7

S8
