# Supplementary material for: SK channel-mediated metabolic escape to glycolysis inhibits ferroptosis and supports stress resistance in C. elegans
Source: Cell Death Dis. 2020 Apr 23;11(4):263. doi: 10.1038/s41419-020-2458-4 (PMC7181639; doi:10.1038/s41419-020-2458-4)
Supplement: Supplementary file 1 — Supplementary figure legends [file 41419_2020_2458_MOESM1_ESM.docx]

**Supplementary figure 1. Erastin kinetics in glucose and galactose** (A and B) MTT assay in HT22 cells treated for 16h with different CyPPA concentrations (5 - 50µM) in the absence or presence of erastin (1.5µM) in conditions of glucose (A) or galactose (B). Data are presented as mean±SD, n=6,^*^*p<*0.05, ^***^*p<*0.001, ^###^*p<*0.001, ^*^compared to control, ^#^compared to erastin alone. (C and E) xCELLigence real-time impedance measurements of HT22 cells challenged with erastin (1.5µM) in the presence of different CyPPA concentrations (1 - 50µM) in glucose (C) or galactose (E). Data are presented as mean±SD, n=6. (D and F) MTT assay in HT22 cells challenged with different erastin concentrations (1µM – 3µM) with or without CyPPA (10µM), in the presence of glucose (D) or galactose (F). Data are presented as mean±SD, n=6, ^***^*p<*0.001, ^##^*p<*0.01, ^###^*p<*0.001, ^*^compared to control ^#^compared to CyPPA alone.

**Supplementary figure 2. BSO treatment in HT22 cells.** MTT assay (A), Annexin/PI measurements (B), xCELLigence real-time impedance measurement (C) and mitochondrial superoxide production measured by mitoSOX fluorescence (D) in HT22 cells treated with different concentrations of BSO (1 - 150µM) in the absence or presence of 10µM CyPPA (in experiments A, B and D). Data are presented as mean±SD, ^***^*p*<0.001 compared to ctr, ^###^*p*<0.001 compared to BSO alone.

**Supplementary figure 3 Mitochondrial morphofunction in HT22 cells.** (A) Image processing of TMRM-stained HT22 cells; original image recorded from the microscope (RAW); Binary image (BIN); Masked image (MSK). (B) Mitochondrial “morphofunction” parameters after 24h (upper panels) or (C) 48 h (lower panels) of CyPPA treatment (1µM) extracted by high-resolution microscopy are shown. Descriptors ‘casum’ and ‘Nn’ are based respectively on Calcein and Hoechst staining. The average mitochondrial intensity of the TMRM signal is represented as ‘density mean’. Data are presented as mean±SD, n=3, ^**^*p<*0.01, ^***^*p<*0.001 versus glucose alone, ^#^*p<*0.05, ^##^*p<*0.01, ^###^*p<*0.001 versus galactose only treated cells.

**Supplementary figure 4. CyPPA kinetics in glucose and galactose** (A and B) MTT assay in HT22 cells treated with CyPPA (5, 10, 25 and 50µM, grey bars) in glucose and galactose treated for 24h (A) and 48h (B). Data are presented as mean±SD, n=6, ^***^*p<*0.001, ^###^*p<*0.001, *compared to glucose control ^#^compared to galactose control.

**Supplementary figure 5. Neuroprotective effects of CyPPA dependent on glycolytic activity** (A and B) Subtracting the basal ECAR values from the maximum ECAR values in response to oligomycin application provided the values of the glycolytic reserve in the left panel (A) for glucose and in the right panel (B) for galactose. No significant differences are observed following CyPPA application. (C) depicts ATP production, calculated by subtracting OCR value after oligomycin application from the OCR value before oligomycin. Data are presented as mean±SD, n=3-6, ^*^*p<*0.05, ^***^*p<*0.001 compared to control. In galactose, no difference in ATP production is observed after CyPPA application. (D) Representative measurement of the normalized oxygen consumption rate (OCR) in glucose (left) and galactose (right) following the application of CyPPA (5 and 10µM, time point of injection indicated by the arrow). (D) glycolytic capacity calculated from ECAR measurements in glucose (left panel) or galactose (right panel) following CyPPA treatment. Data are presented as mean±SD, n=3-6 per condition.

**Supplementary figure 6. Neuroprotective effects of CyPPA dependent on glycolytic activity** (A – F) MTT assay in HT22 cells treated with different concentrations of DCA (1 – 10mM (A and D) for 24h. (B and E) HT22 cells treated with DCA before (8h) and during erastin (1.5µM, 16h) stimulation. (C and F) HT22 cells treated 8h with DCA and CyPPA (10µM) before and during erastin (1.5µM, 16h) stimulation. (A – C) represent experiments of cells grown in glucose, (D – F) represent experiments of cells grown in galactose. Data are presented as mean±SD, n=6, ^*^*p<*0.05, ^***^*p<*0.001 compared to control, ^###^*p<*0.001 compared to erastin alone, ^$$$^*p<*0.001 compared to erastin + CyPPA. (G) MTT assay of HT22 cells pre-treated for 6h with lactate (0-150mM) and challenged with erastin (1.5μM) for 16h.

**Supplementary figure 7. Neuroprotective effects of CyPPA dependent on mitochondrial ROS production** (A) Represents maximum uncoupled respiration, measured by high-resolution respirometry, obtained after titration with FCCP. (B) Mitochondrial membrane potential loss was measured using the fluorescent dye TMRE and FACS analysis after CyPPA (10-50µM, 24h) exposure and 30 min CCCP treatment as positive control (50µM). Data are presented as mean±SD, n=3 ^*^*p<*0.05, ^**^*p<*0.01, ^***^*p<*0.001 compared to control. (C) Mitochondrial superoxide levels were measured using the fluorescent dye MitoSOX and FACS analysis after MnTBAP treatment (5 - 20µM) for 24h in either glucose or galactose. Data are presented as mean±SD, n=3 ^*^*p<*0.05, ^**^*p<*0.01, ^***^*p<*0.001 compared to control, ^##^*p<*0.01, ^###^*p<*0.001 compared to glucose alone. (D – J) MTT assay in HT22 cells treated with MnTBAP (different concentrations, 1-20µM) without (D, glucose; G, galactose) and before and during erastin challenge (1.5µM) (E and I) or only during erastin challenge (H). (F and J) MTT assay after treatment of 8h MnTBAP in the presence of CyPPA, following treatment in the presence of erastin (1.5µM). Data are presented as mean±SD, n=6, ^*^*p<*0.05, ^**^*p<*0.01, ^***^*p<*0.001 compared control, ^#^*p<*0.05, ^##^*p<*0.01, ^###^*p<*0.001 compared to erastin treatment, ^$^*p<*0.05, ^$$^*p<*0.01, ^$$$^*p<*0.001 compared to erastin + CyPPA.

**Supplementary figure 8. SK channel-mediated protection against paraquat in HT22 cells** (A) MTT assay in HT22 cells after paraquat (PQ) treatment (500 – 1000µM) (24h) in the absence or presence of CyPPA (10 or 50µM). (B) MTT assay in HT22 cells after paraquat treatment (350-450µM) (24h) in the absence or presence of CyPPA (10 or 50µM). Data are presented as mean±SD, n=6, ^***^*p<*0.001 compared control, ^##^*p<*0.01, ^###^*p<*0.001 compared to corresponding PQ treatment. (C) Representative measurement of the normalized oxygen consumption rate (OCR) in HT22 cells following 6 h treatment of CyPPA (10µM). Injections are indicated by the arrows, A; oligomycin (4µM), B; dinitrophenol (DNP) (50µM), C; rotenone (150nM) and antimycin A (1µM), D; 2-DG (50mM). Data are presented as mean±SD, n=3-6 per condition.
