# Supplementary figures and images for: SK channel-mediated metabolic escape to glycolysis inhibits ferroptosis and supports stress resistance in C. elegans
Source: Cell Death Dis. 2020 Apr 23;11(4):263. doi: 10.1038/s41419-020-2458-4 (PMC7181639; doi:10.1038/s41419-020-2458-4)

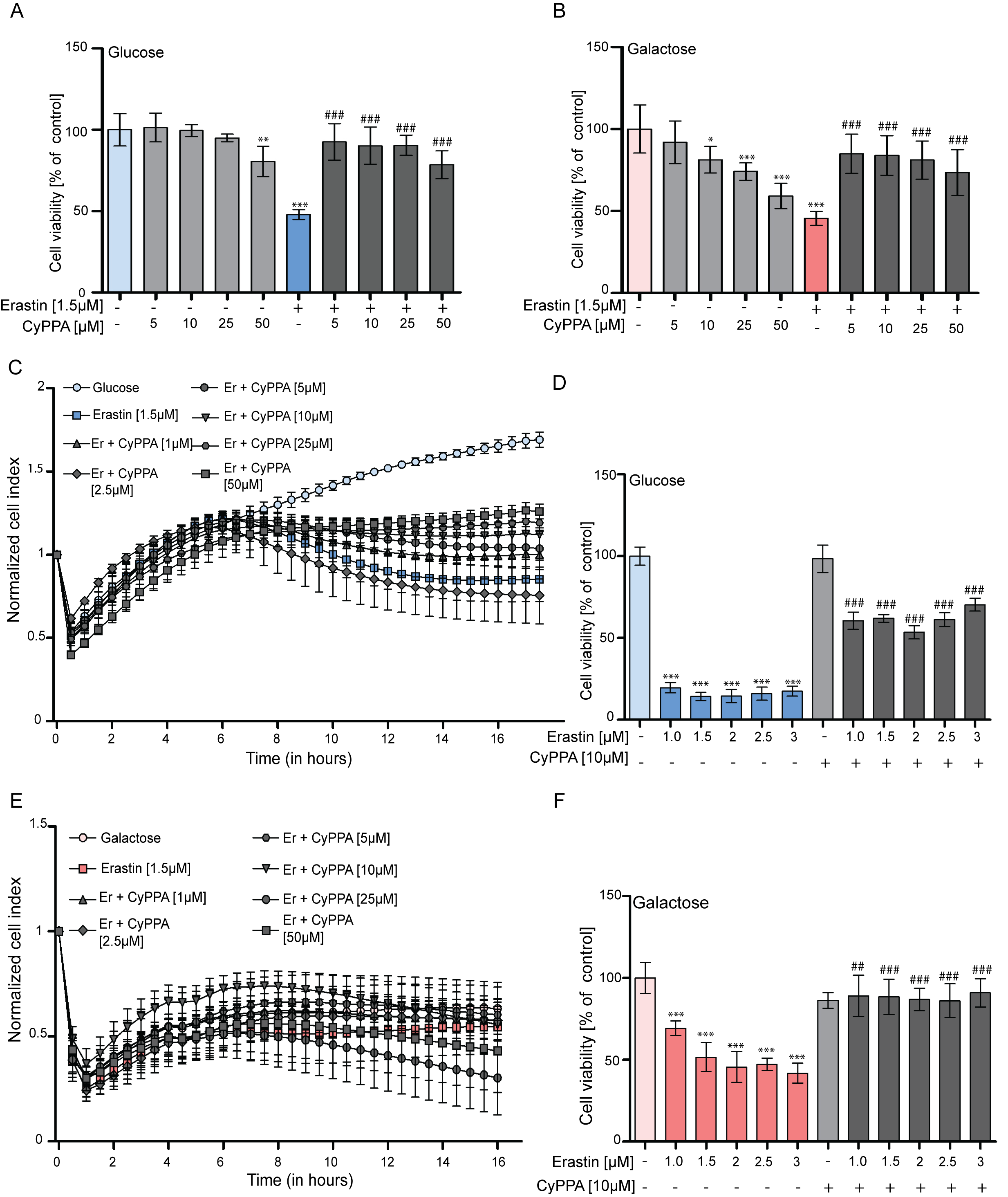

Supplement: Supplementary file 2 — S1 [file 41419_2020_2458_MOESM2_ESM.tif]

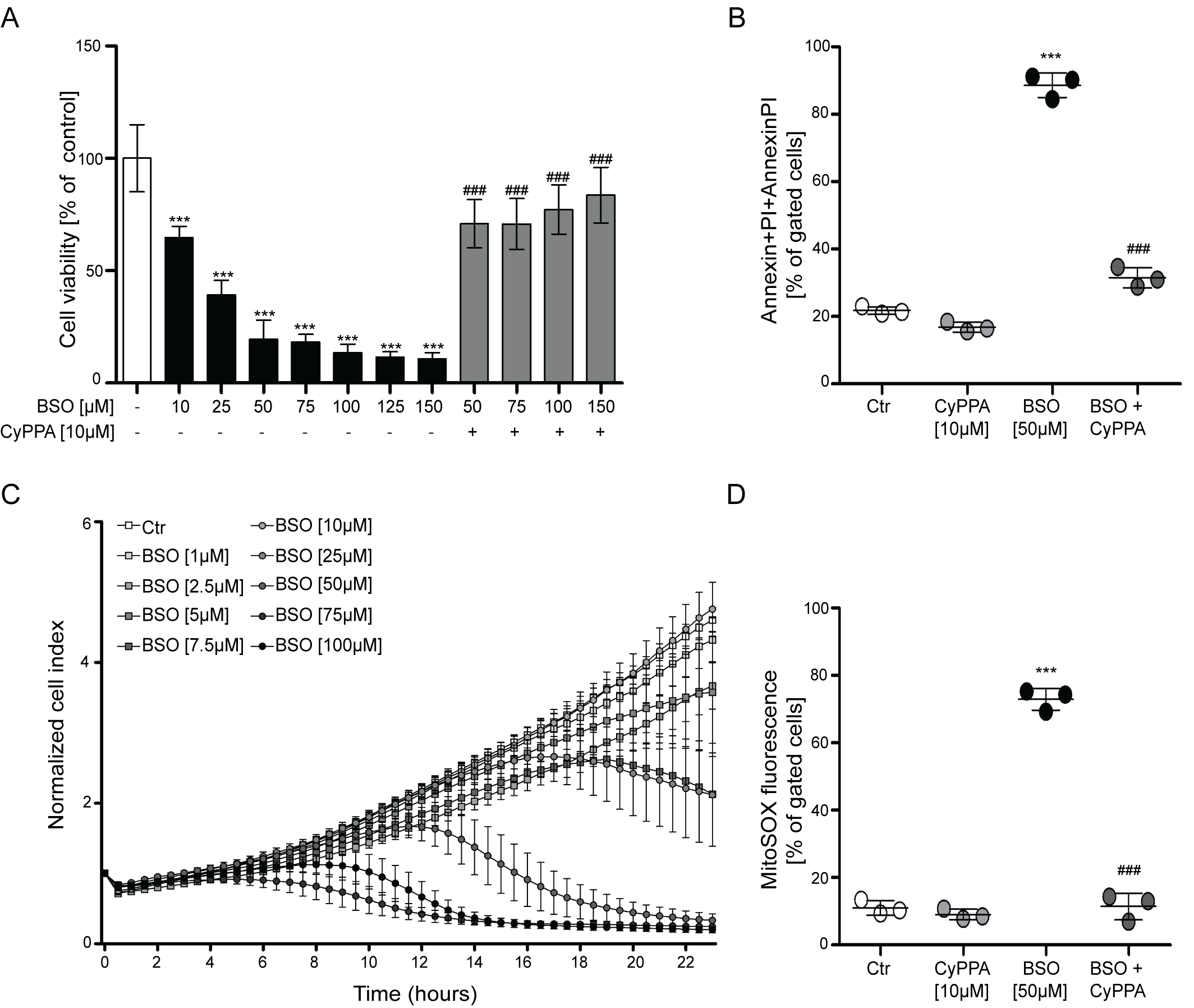

Supplement: Supplementary file 3 — S2 [file 41419_2020_2458_MOESM3_ESM.tif]

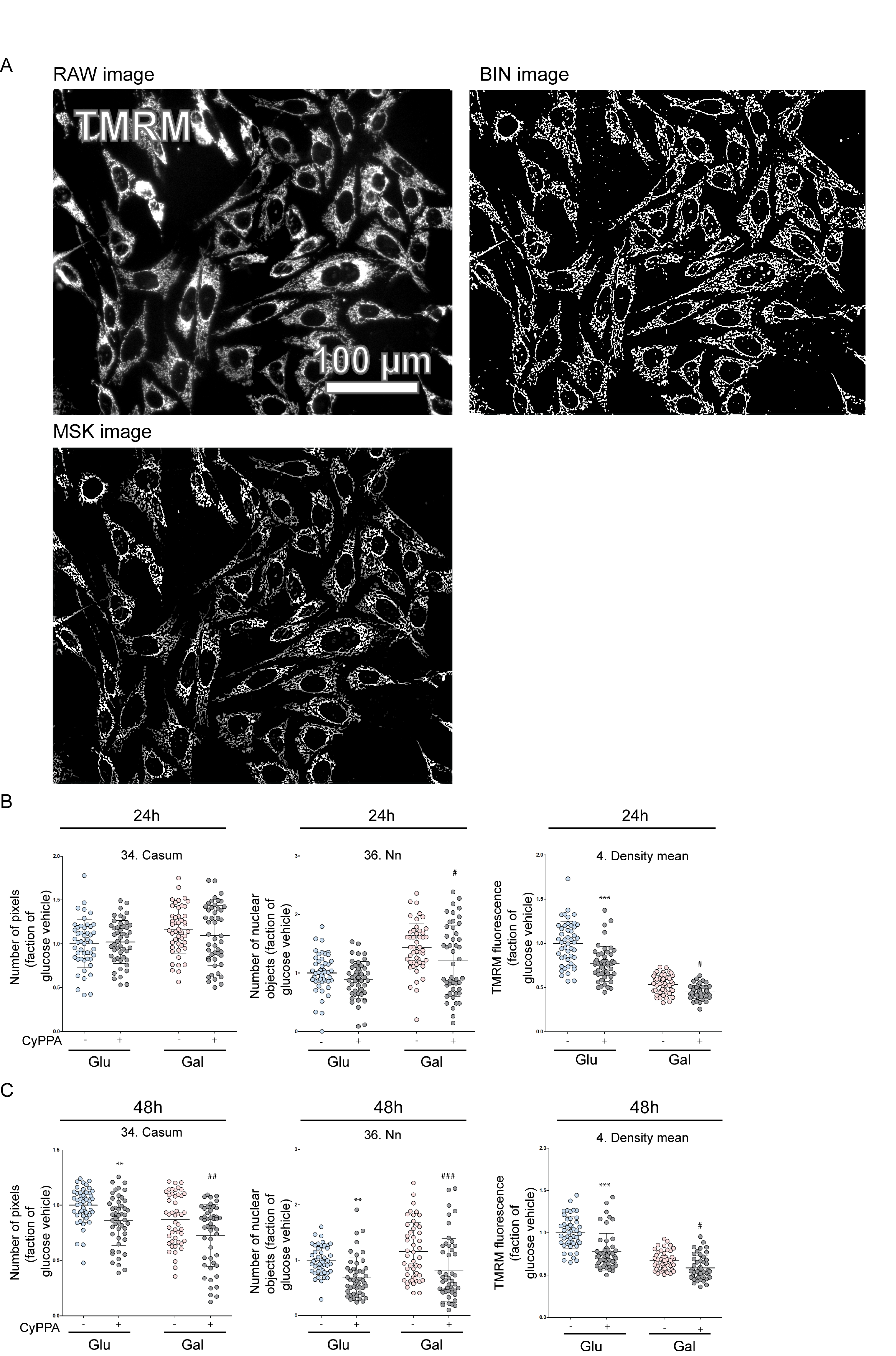

Supplement: Supplementary file 4 — S3 [file 41419_2020_2458_MOESM4_ESM.tif]

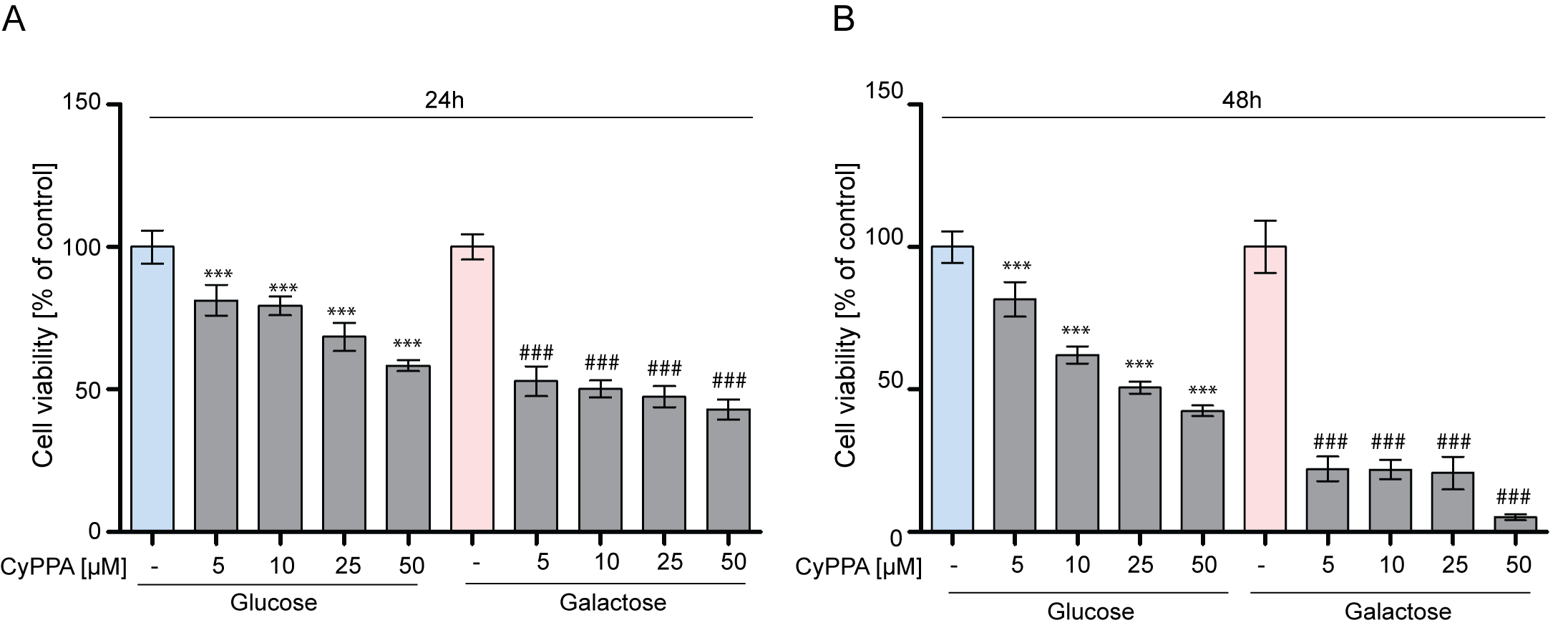

Supplement: Supplementary file 5 — S4 [file 41419_2020_2458_MOESM5_ESM.tif]

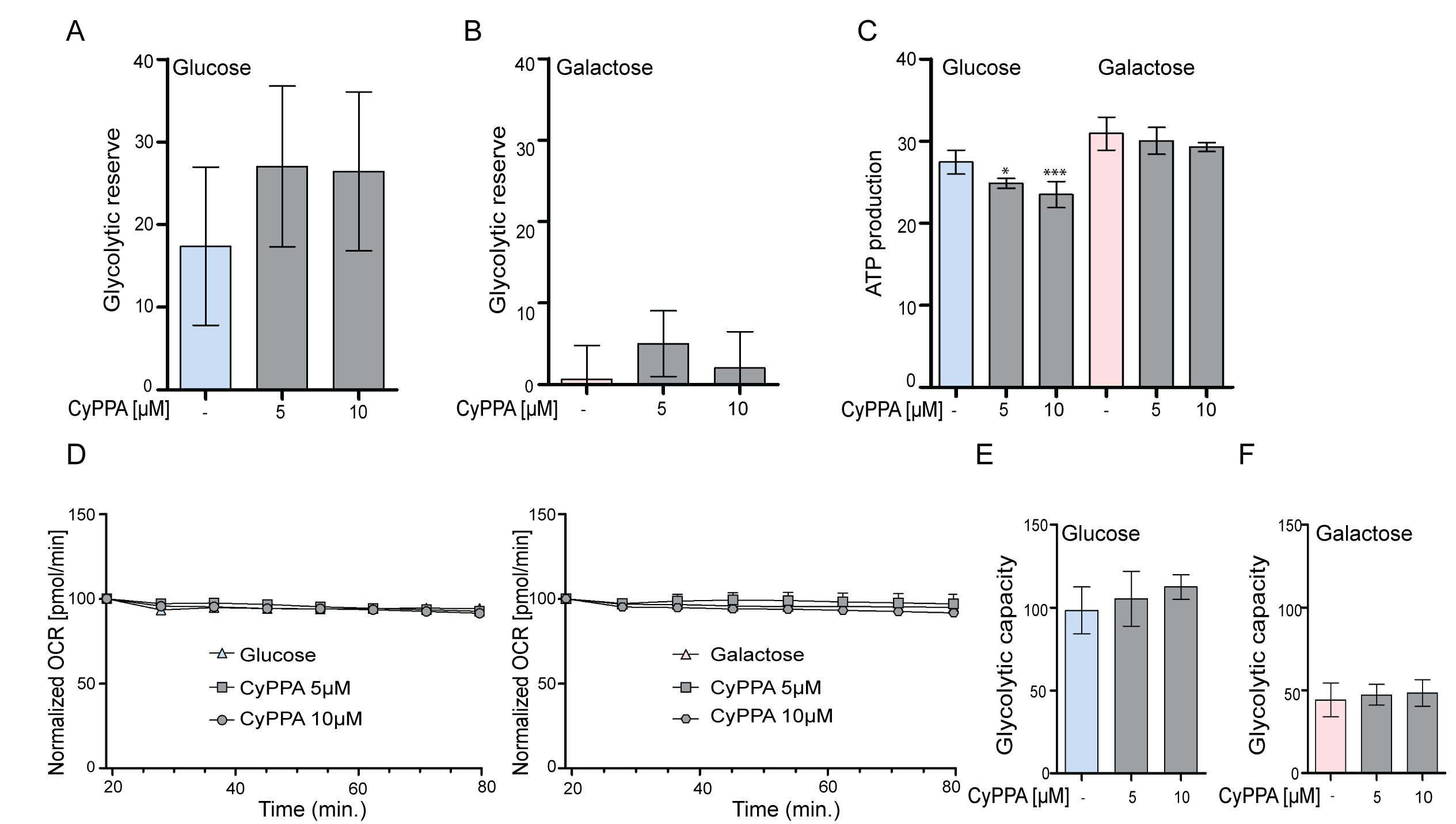

Supplement: Supplementary file 6 — S5 [file 41419_2020_2458_MOESM6_ESM.tif]

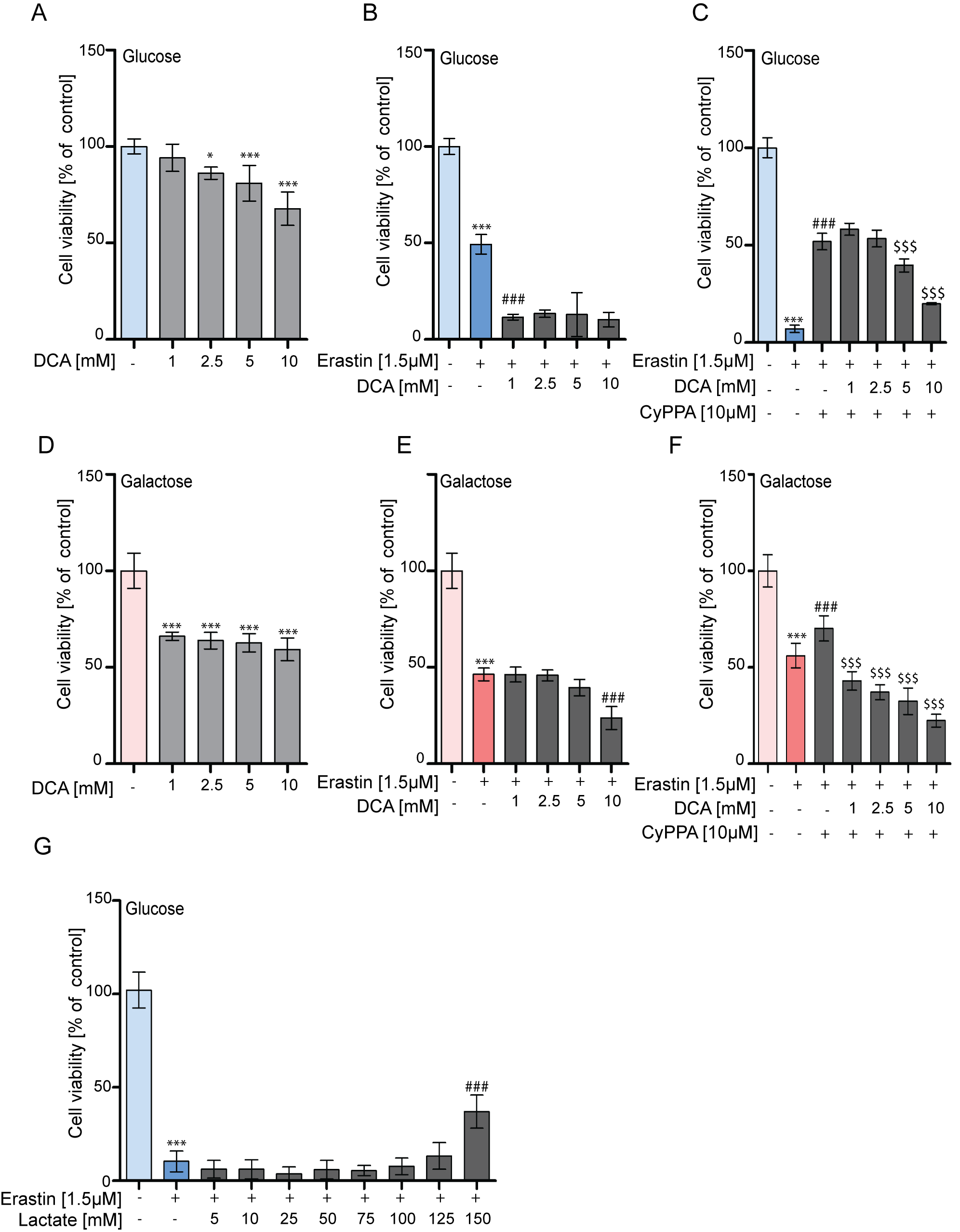

Supplement: Supplementary file 7 — S6 [file 41419_2020_2458_MOESM7_ESM.tif]

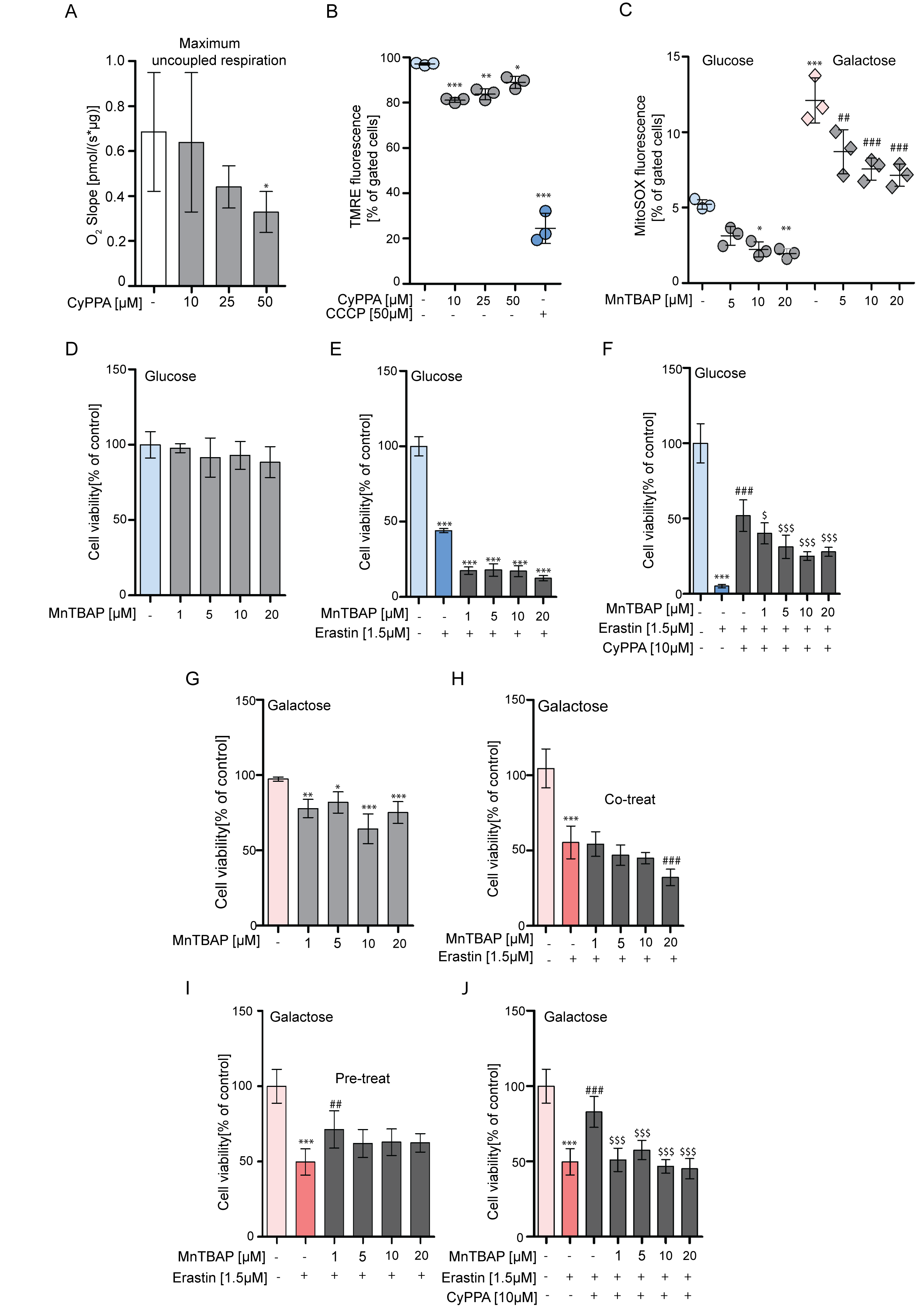

Supplement: Supplementary file 8 — S7 [file 41419_2020_2458_MOESM8_ESM.tif]

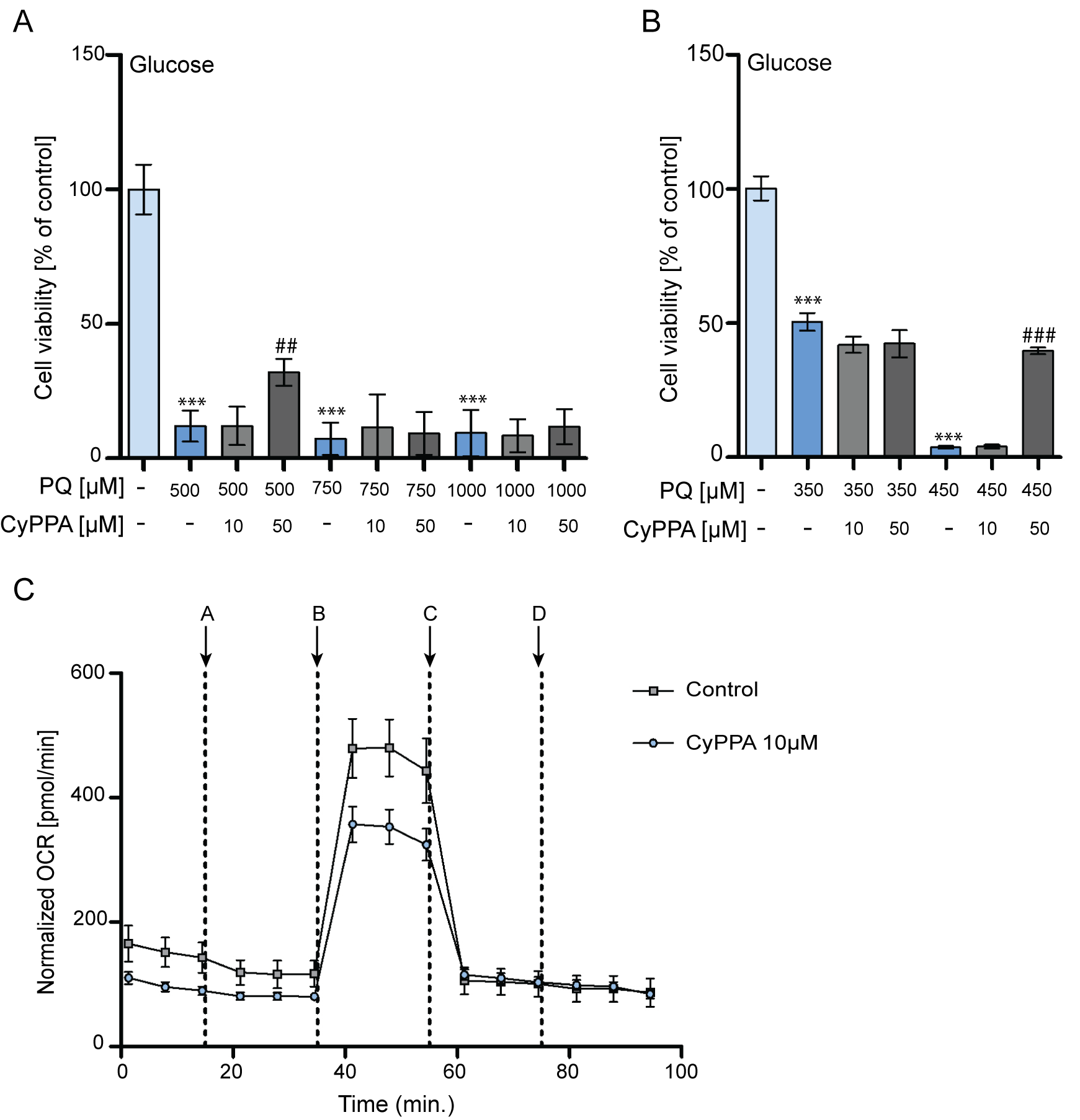

Supplement: Supplementary file 9 — S8 [file 41419_2020_2458_MOESM9_ESM.tif]
